# Ventricular voltage‐gated ion channels: Detection, characteristics, mechanisms, and drug safety evaluation

**DOI:** 10.1002/ctm2.530

**Published:** 2021-10-14

**Authors:** Lulan Chen, Yue He, Xiangdong Wang, Junbo Ge, Hua Li

**Affiliations:** ^1^ Department of Cardiology, Shanghai Institute of Cardiovascular Diseases Shanghai Xuhui District Central Hospital & Zhongshan‐xuhui Hospital, Zhongshan Hospital, Fudan University Shanghai China; ^2^ Department of Cardiology Shanghai Xuhui District Central Hospital & Zhongshan‐xuhui Hospital Shanghai China; ^3^ Institute of Clinical Science, Zhongshan Hospital Fudan University Shanghai China

**Keywords:** action potentials, cardiac voltage‐gated ion channel, cardiovascular safety evaluation, electrophysiological techniques

## Abstract

Cardiac voltage‐gated ion channels (VGICs) play critical roles in mediating cardiac electrophysiological signals, such as action potentials, to maintain normal heart excitability and contraction. Inherited or acquired alterations in the structure, expression, or function of VGICs, as well as VGIC‐related side effects of pharmaceutical drug delivery can result in abnormal cellular electrophysiological processes that induce life‐threatening cardiac arrhythmias or even sudden cardiac death. Hence, to reduce possible heart‐related risks, VGICs must be acknowledged as important targets in drug discovery and safety studies related to cardiac disease. In this review, we first summarize the development and application of electrophysiological techniques that are employed in cardiac VGIC studies alone or in combination with other techniques such as cryoelectron microscopy, optical imaging and optogenetics. Subsequently, we describe the characteristics, structure, mechanisms, and functions of various well‐studied VGICs in ventricular myocytes and analyze their roles in and contributions to both physiological cardiac excitability and inherited cardiac diseases. Finally, we address the implications of the structure and function of ventricular VGICs for drug safety evaluation. In summary, multidisciplinary studies on VGICs help researchers discover potential targets of VGICs and novel VGICs in heart, enrich their knowledge of the properties and functions, determine the operation mechanisms of pathological VGICs, and introduce groundbreaking trends in drug therapy strategies, and drug safety evaluation.

## Graphical abstract

1. The structure, distribution, interaction and function of voltage‐gated ion channels (VGICs) maintains a physiological balance of the ionic currents, normal action potential (AP), excitation‐contraction coupling, and synchronization in cardiomyocytes.
2. Various genetic mutations and molecular dysregulation of the VGICs could result in ionic imbalance, abnormal AP waveform, and even cardiac arrhythmia.

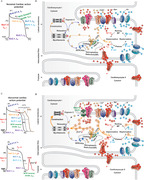

## 1 INTRODUCTION

The cardiac cycle begins when an action potential (APs) is spontaneously generated in the sinoatrial node (SAN), the primary pacemaker in the heart. The coordinated propagation of synchronized electrical impulses relies on effective cooperation among various components in the heart system to maintain cardiac rhythm.^1^ Specifically, the AP from the SAN passes sequentially through the atria, the atrioventricular node (AVN), and His‐Purkinje conducting tissue before ultimately reaching the ventricles.^1^ APs, which are generated and modulated by the opening and closing of ion channels in the plasma membrane, are the fundamental electrical excitation signals responsible for the beating of cardiomyocytes and are distinct among various components in the heart due to various expression of ion channels.^1^ Among the voltage‐gated ion channels (VGICs) involved in ventricular APs, sodium (Na^+^), potassium (K^+^), and calcium (Ca^2+^) channels (Nav, Kv, and Cav channels, respectively) predominate.^2^ The functions of VGICs, the mechanisms underlying cardiac physiology and pathology, and appropriate diagnostic and treatment strategies have been explored for decades through electrophysiological techniques. Moreover, these techniques have been developed and expanded over time; from laborious, low‐throughput methods limited to whole‐cell experiments, they have been refined into automated, high‐throughput methods. These developments have dramatically augmented the ability of researchers to further explore VGICs. In the real world, aside from genetic mutations affecting VGICs, many drugs can bind to these channels, block ion flow and disrupt the regulation of APs, potentially leading to drug‐induced arrhythmia, or “proarrhythmia.”^3^, ^4^ It is necessary to evaluate the risks of potential drug candidates by using the different techniques mentioned above,^5^, ^6^ according to the US Food and Drug Administration (FDA) guidelines. In this respect, enhancing the quality of preclinical safety screening is particularly important for validating the safety of therapies to avoid potential adverse effects on ion channels and prevent billions of dollars in losses because of late‐stage premarket drug withdrawals in the development process of drug development before marketing.

## 2 METHODS FOR DETECTING CARDIAC VGICS

### 2.1 Electrophysiological techniques

The manual patch clamp (MPC) technique (Table S1) is the gold standard for analyzing electrophysiological characteristics (APs and specific ion channel currents) in cardiac myocyte research studies under physiological/pathological conditions or in response to drug application. Three main cell models are used: 1) freshly isolated ventricular myocytes from wild‐type (WT), diseased or genetically modified animal models; 2) heterologous expression systems specifically expressing the human ion channels of interest; and 3) human induced pluripotent stem cell‐derived cardiomyocytes (hiPSC‐CMs) from healthy individuals and patients.^2^, ^7^ In addition, cardiac ion channels can be examined by single‐channel MPC recording (Table S1); for example, this technique can be applied to a potential new channel with a putative pore‐containing structure,^8^ or channels that cannot be expressed or trafficked on the cell membrane in heterologous expression systems,^9^ or channels that are potentially altered in the diseased heart.^9^ The main limitation of MPC is its low throughput. Therefore, the automated patch clamp (APC) (Table S1) enables much higher‐throughput experiments while nevertheless achieving high‐quality seals, thereby facilitating the use of the MPC and is now routinely used in cardiac drug discovery and safety testing.^10^, ^11^, ^12^

In addition, microelectrode arrays (MEAs) (Table S1) offer an alternative noninvasive that enables noninvasive, high‐throughput assays evaluating extracellular field potential (EFP) of excitable cells^13^; MEAs have also been increasingly used in cardiology to test the safety of drug candidates.^13^, ^14^ Furthermore, impedance techniques (Table S1) have recently been combined with EFP recording on the same platform to provide a noninvasive, high‐throughput and long‐term measurement strategy for assessing the synchronous beating of monolayer cardiomyocytes, the duration of EFPs, and the proarrhythmogenic capacity of drug candidates without altering cellular physiology^15^; this combined approach offers a more comprehensive analysis of excitation‐contraction (EC) coupling than either component alone.

Generally, low‐throughput MPC is a critical tool for examining the electrophysiological characteristics of cardiac cells and the biophysical properties and functions of ion channels. APC and MEAs have developed into an indispensable platform for pharmaceutical companies and academic laboratories to conduct potential drug target discovery, drug screening, and cardiac safety with high efficiency and accuracy. hiPSC‐CM‐based APC,^16^ MEA,^17^ and impedance^15^ screening assays are increasingly used to evaluate antiarrhythmic effects, adverse effects or interindividual variations in patients or healthy individuals and to acquire more comprehensive validation data.

### 2.2 Joint techniques

Cryoelectron microscopy (cryo‐EM) (Table S2), which can resolve the structure of macromolecular complexes at the atomic conformation level, has provided researchers with a more in‐depth molecular picture of ion selectivity, voltage gating, and intersubunit interactions in channel complexes and thereby provides insights into important biological phenomena, such as electrophysiological feature variations among different VGIC isoforms,^18^, ^19^ feature changes after the application of various compounds,^20^ and the mechanisms of mutation‐related arrhythmia.^18^ Hundreds of disease‐associated missense mutations have been mapped onto all major domains in the structure of many VGICs.^18^, ^21^ Cryo‐EM structure analysis could provide novel insights into both VGIC‐drug interactions and the mechanisms of action of such drugs.^20^, ^22^ Moreover, electrophysiological techniques can help evaluate whether the functional properties of truncated or mutated VGICs purified for cryo‐EM analysis are similar to those of WT full‐length VGICs.^18^, ^19^

Optical imaging methods (Table S2) using voltage‐ or Ca^2+^‐sensitive dyes are less invasive than MPC and are able to measure changes in the MPs, intracellular calcium concentrations, electrical activity and EC coupling of cardiac cells.^23^ However, some sensitive dyes are limited by cytotoxicity and short half‐lives. Genetically encoded fluorescent Ca^2+^ indicators, such as ArcLight and GCaMP, were developed and applied to cardiac research to monitor functional changes in hiPSC‐CMs in a long‐term, noninvasive, high‐throughput manner.^24^, ^25^ The combination of optical imaging and electrophysiological techniques allows simultaneous recording of optical AP signals and calcium transient signals and permits both high spatial resolution and accurate functional evaluation.

Optogenetics approaches (Table S2), using light to control the perturbation of membrane voltage through the opening of optogenetic channels have been used to modulate cardiomyocyte excitability and heart rate with high precision and to explore the mechanisms of arrhythmia generation.^26^, ^27^, ^28^ Optogenetic channels can also be used to study the relationship between cardiac myocytes and nonmyocyte cells and provide a feasible way to explore direct evidence of electrical coupling between these cells in normal or injured regions of the heart.^29^ Automated frequency‐dependent cardiotoxicity screening can be conducted by applying optogenetic stimulation similar to physiological heart rates in hiPSC‐derived cardiomyocytes.^30^

## 3 AP GENERATION AND EC COUPLING OF CARDIOMYOCYTES

### 3.1 Normal electrophysiology of AP and EC coupling

**FIGURE 1:**
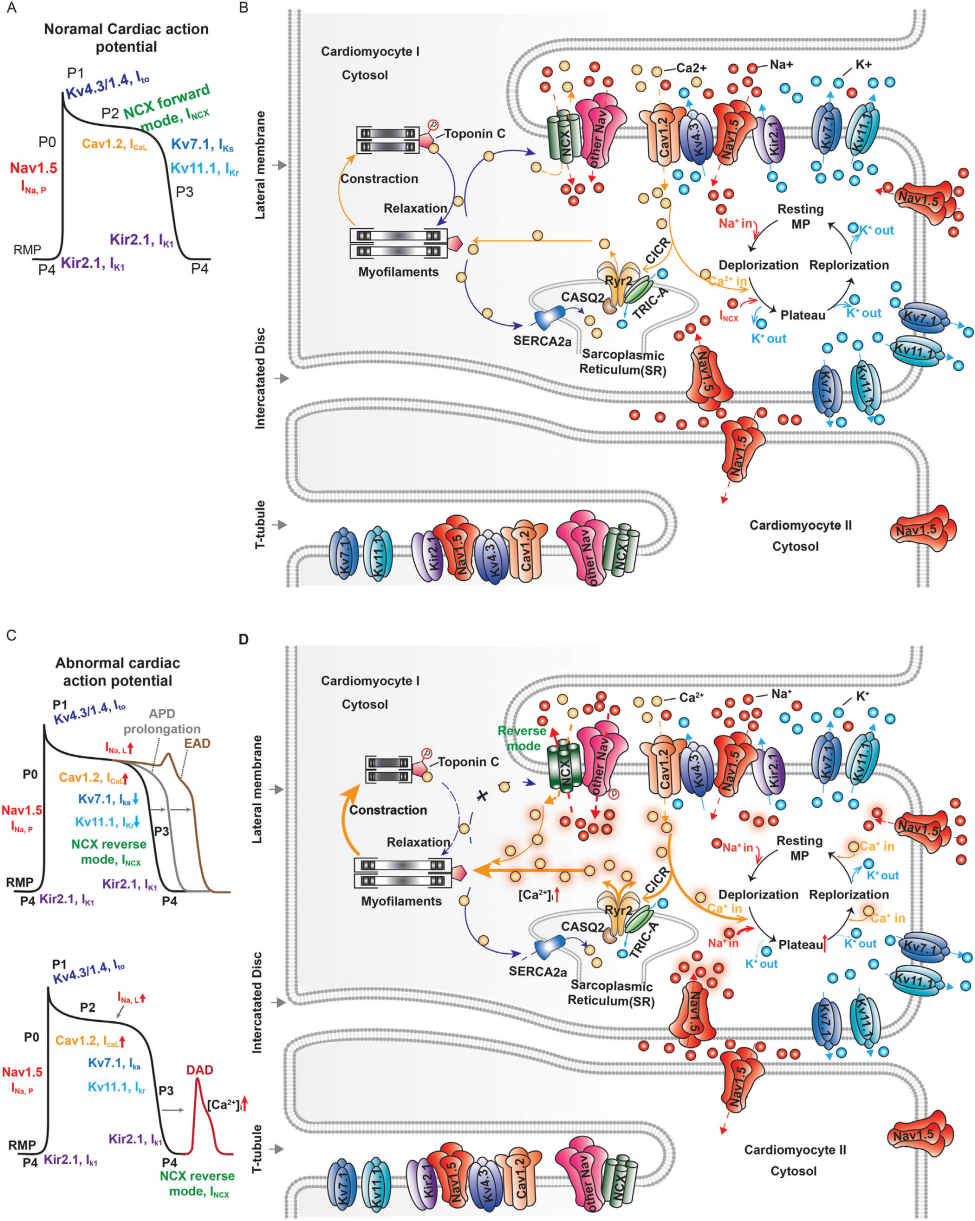
Normal AP generation and EC coupling of cardiomyocytes; abnormal electrophysiology as a trigger of arrhythmias. (A) A typical ventricular action potential (AP) and the depolarizing and repolarizing ionic currents underlying its different phases (P0‐P4). (B) Voltage‐gated ion channel (VGIC) distribution and contribution to AP excitation‐contraction coupling in cardiomyocytes. Na^+^, Ca^2+^, and K^+^ are represented by red, yellow, and blue dots, respectively. The cardiac VGICs Nav1.5, Kv7.1, and Kv11.1 are primarily localized in intercalated discs (IDs), T‐tubules and lateral membranes. TTX‐sensitive Nav channels are primarily localized in T‐tubules and colocalized with the Na/Ca exchanger (NCX). The sarcoplasmic reticulum (SR) channel ryanodine receptor 2 (RyR2) is located near most L‐type Ca^2+^ channels, and Cav1.2 is located in T‐tubules. RyR2 is regulated by type 2 calsequestrin (CASQ2). A novel TRIC‐A channel can also directly interact with RyR2 and act as a counterion channel to modulate Ca^2+^ release from the SR. The potassium channel Kir2.1 interacts with Nav1.5 in T‐tubules. Kir2.1‐mediated outward K^+^ drives repolarization, while the rapid increase in membrane potential (MP) depolarization and the MP overshoots during Phase I are driven by the influx of Na^+^, which is mediated by Nav1.5. Then, Kv4.3‐ and Kv1.4‐mediated fast and slow transient outward K^+^ currents (I_to, fast_ and I_to, slow_) are activated to mediate partial repolarization in Phase I. During the plateau phase (Phase II), nearly equal inward currents are mediated by Cav1.2 (Ca^2+^ in) and the NCX in forward mode (3 Na^+^ in, 1 Ca^2+^ out), while outward currents (K^+^ out) are mediated by the voltage‐gated delayed rectifier potassium channels Kv7.1 and Kv11.1. In addition, Ca^2+^ influx mediated by Cav1.2 activates RyR2 channels to open, thereby releasing additional Ca^2+^ into the cytosol via a process known as Ca^2+^‐induced Ca^2+^release (CICR). This process induces the Ca^2+^ sensing protein troponin C on myofilaments to begin to contract. During late Phase II and Phase III, Kv7.1‐ and Kv11.1‐mediated outward currents (K^+^ out) become dominant, resulting in repolarization, until they are again surpassed by Kir2.1 activity, resulting in the maintenance of repolarization at the resting MP during Phase IV. Cytosolic Ca^2+^ flows back into the SR via Ca^2+^‐ATPase type‐2a (SERCA2) and back to the extracellular space via the NCX. Contraction is terminated when cytosolic Ca^2+^ levels fall below the level required for the Ca^2+^‐troponin association (resulting in dissociation). (C) Abnormal ventricular APs. A prolonged AP duration (APD, in gray) due to an abnormal increase in the inward current (I_Na,L_ and I_CaL_) and a decrease in the outward current (I_K_) can develop into an arrhythmia trigger called early afterdepolarizations (EADs) (in brown) during the plateau phase (upper). Another arrhythmia trigger called delayed afterdepolarizations (DADs) (in red) occurs due to cytosolic Ca^2+^ overload during the diastole period (lower). (D) An abnormal increase in the Na^+^ current (represented by red sparkling dots) mediated by Nav1.5 and other Nav channels then induce further depolarizing plateau currents by reactivating the inward I_CaL_ (represented by yellow sparkling dots), and an abnormal decrease in K^+^ out results in a prolonged plateau phase. This abnormal Na^+^ accumulation switches the NCX to reverse mode, in which it pumps 3Na^+^ out of the cell while transferring Ca^2+^ into the cytosol. A further increase in the Ca^2+^ concentration prolongs repolarization and enhances excitation‐contraction coupling. During the diastole period, abnormal release of Ca^2+^ via the reopening of RyR2 and influx of Ca^2+^ via reverse‐mode NCX activity give rise to Ca^2+^ overload in the cytosol, resulting in DADs

A typical ventricular AP consists of five phases (P0‐P4) that are mediated by different depolarizing and repolarizing ionic currents (Figure 1A).^2^ The initial phase (Phase 0) of a cardiac AP occurs after the resting state (Phase IV) of the previous AP and arises from a very large inward I_Na_ current mediated by Nav channels. Then, Kv channels are activated to mediate transient outward potassium currents I_to_, leading to partial repolarization in Phase I. During Phase II, L‐type Cav channels (LTCCs) are activated, generating an inward I_CaL_ current. In addition, the Na^+^/Ca^2+^‐exchanger (NCX) opens in forward mode and mediates an inward I_NCX_ current by exchanging an influx of 3Na^+^ for an efflux of 1Ca^2+^. On the other hand, the voltage‐gated delayed rectifier potassium channels open and mediate outward rectifier currents (I_Kr_ and I_Ks_). Membrane potential (MP) changes extraordinarily little due to the nearly equal inward and outward currents during this phase, which is also known as the plateau phase. In the late plateau phase, LTCCs are inactivated, and the dominant outward currents, I_Kr_ and I_Ks_, result in repolarization in Phase III. Toward the end of Phase III, I_Kr_ and I_Ks_ decline, and the inwardly rectifying potassium channels Kir2.x mediate the I_K1_ current to drive repolarization and maintain a resting MP (Phase IV).

The beating of the heart relies on EC coupling (Figure 1B). During AP generation, the LTCC‐mediated increase in the cytosolic Ca^2+^ concentration instantaneously triggers the opening of the ryanodine receptor 2 (RyR2) channel, a Ca^2+^ channel in the sarcoplasmic reticulum (SR), which causes Ca^2+^ release from the SR, and thereby further increases the cytosolic Ca^2+^ concentration. This Ca^2+^‐induced Ca^2+^‐release (CICR) prompts Ca^2+^‐sensing protein troponin C to initiate contraction (systole). Cytosolic calcium levels are reduced via the Ca^2+^‐ATPase type‐2a (SERCA2)‐mediated influx of Ca^2+^ back into the SR and the NCX‐mediated efflux of Ca^2+^ back to the extracellular space, resulting in the dissociation of calcium and troponin and then muscle relaxation (diastole).^2^, ^31^, ^32^

### 3.2 Abnormal electrophysiology as a trigger of arrhythmias

Disruption of the normal generation and duration of Aps is associated with arrhythmias in the heart.^1^, ^31^ Two types of afterdepolarizations, early afterdepolarizations (EADs) and delayed afterdepolarizations (DADs), could induce premature APs and contribute to arrhythmias. EADs occurs during Phase II or III (Figure 1C). Prolongation of action potential duration (APD) due to the reduction of repolarization currents (eg, I_Kr_ and I_Ks_) or the increase in I_Na,L_ current could give rise to abnormal recovery from the inactivation of LTCC channels and further depolarize the membrane due to the reactivation of inward currents I_CaL_.^1^, ^2^ DADs can result from depolarization after the end of AP repolarization (Figure 1C), potentially due to Ca^2+^ overload caused by enhanced SR Ca^2+^ release and the inappropriate activation of the reverse mode of NCX, which mediates outward I_NCX_ current by exchanging an influx of 1Ca^2+^ for an efflux of 3Na^+^.^1^, ^2^

Under physiological conditions, Nav channel activity is regulated by cytosolic Ca^2+^ levels, such as elevation of cytosolic Ca^2+^ levels resulting in destabilization of inactivation and increase of the amount of available channels to open for the next AP.^33^, ^34^, ^35^ And Na^+^ influx, in turn, affects the modulation of cytosolic Ca^2+^ levels.^32^ However, under pathological conditions, an abnormal increase in Na^+^ during diastole can result in inappropriate timing of reverse flow through the NCX channel (3Na^+^ efflux and 1Ca^2+^ influx), further increasing the cytosolic Ca^2+^ concentration and altering normal EC coupling (Figure 1D).^32^

## 4 VENTRICULAR AP‐RELATED ION CHANNELS: CLASSIFICATION, STRUCTURE, FUNCTION, REGULATION, AND DISEASE RELEVANCE

### 4.1 Nav channels

Cardiac voltage‐gated Nav channels initiate AP in electrically excitable cells. The specificities among isoforms (Table 1) are attributed to the distinct α‐subunit encoded by the corresponding gene and the different combinations of β subunits.^36^ β subunits regulate channel surface expression, voltage dependence and gating kinetics.^36^ *SCN5A*‐encoded Nav1.5 is the most abundantly expressed Nav channel in ventricle and atrium (Table 1) and is responsible for the generation of APs and the conduction of cardiac impulses in cardiomyocytes.^18^, ^34^, ^37^, ^38^ Additional evidence has shown that other isoforms are also expressed in the ventricular myocytes (Table 1).^32^, ^39^, ^40^, ^41^, ^42^, ^43^, ^44^, ^45^, ^46^, ^47^, ^48^

**TABLE 1:** Cardiac voltage‐gated Na^+^ channels subtypes

Subtypes	Encoding α subunits Gene	Auxiliary subunits	Main location	Subcellular localization in cardiac tissue (V/A/SAN) and region^37^, ^38^, ^43^, ^233^	Cryo‐EM structure	TTX sensitivity	Principal physiological functions in human ventricle myocytes
Nav1.1	*SCN1A*	β4 encoded by *SCN4B*	CNS, Heart	V≈A≈SAN; T‐tubules	Human Nav1.1‐β4 channel^21^	Sensitive	Cardiac pacemaking and promotes Ca^2+^ dynamics
Nav1.2	*SCN2A*	β2 encoded by *SCN2B*	CNS, Heart	V≈A<SAN T‐tubules	Human Nav1.2‐β2 subunit^52^	Sensitive	Contributes small portion to cardiac sodium current
Nav1.3	*SCN3A*	NR	CNS, Heart	V≈A<SAN T‐tubules	NR	Sensitive	Contributes small portion to cardiac sodium current
Nav1.4	*SCN4A*	β1 encoded by *SCN1B*	Skeletal muscle, Heart	V≈A≧SAN T‐tubules	Electric eel,^234^ human^53^ Nav1.4‐β1 subunit	Sensitive	Contributes small portion to cardiac sodium current
Nav1.5	*SCN5A*	β1, β2 encoded by *SCN1B and SCN2B, respectively*	Heart	V≈A≧SAN; IDs, lateral membrane, T‐tubules	Rabbit Nav1.5 α ‐ β1, β2 subunits^18^	Resisitant	Mediates the entry of Na+, and triggers overshooting of AP
Nav1.6	*SCN8A*	β1 encoded by *SCN1B*	CNS, PNS, Heart	V≈A T‐tubules	NR	Sensitive	Contributes small portion to cardiac sodium current; Promote Ca^2+^ dynamics
Nav1.8	*SCN10A*	β2 encoded by *SCN2B*	PNS, Heart	V<A T‐tubules	NR	Resisitant	Cardiac contraction and conduction

CNS, central nervous system; PNS, peripheral nervous system; IDs, intercalated discs; T‐tubules, transverse tubules; Cryo‐EM, cryoelectron microscopy; TTX, tetrodotoxin; V, ventricle; A, atrium; SAN, sinoatrial Node; NA: not available; NR, not reported.

#### 4.1.1 Nav1.5

**FIGURE 2:**
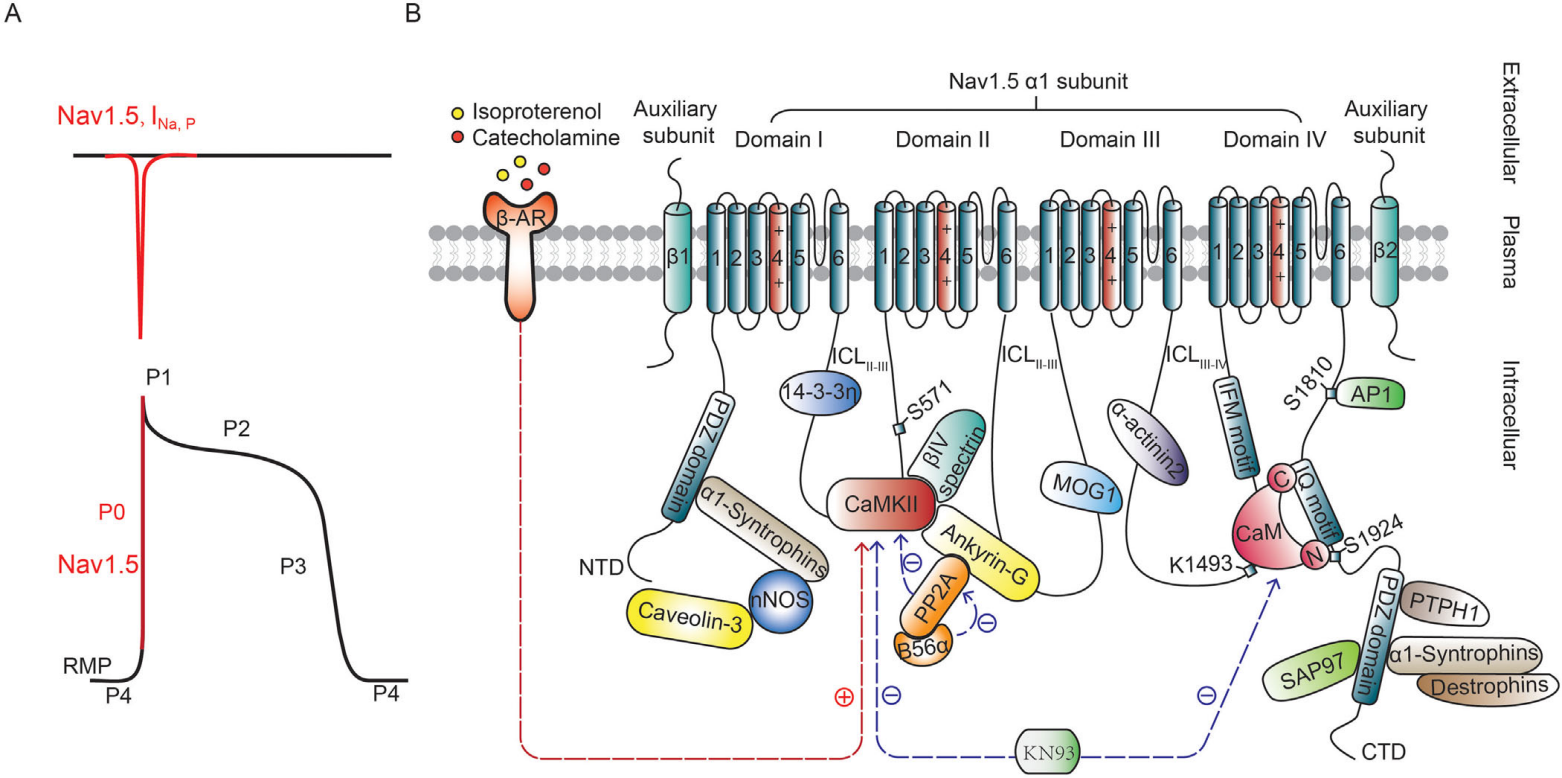
Cardiac voltage‐gated sodium channel (Nav1.5) structure, accessory proteins and signaling. (A) The contribution of I_Na,P_ (upper) mediated by Nav1.5 to action potential (AP) Phase 0 (lower). (B) The cardiac voltage‐gated sodium channel Nav1.5 comprises one α subunit and one or two auxiliary β subunits. The α subunit consists of four homologous but nonidentical repeats (DI‐DIV) connected by cytoplasmic linkers (ICL_I‐II_, ICL_II‐III_, and ICL_III‐IV_) and is responsible for voltage dependence, pore formation, and surface expression. Each domain contains S1‐S6 subunits that are connected by loops located intra‐ and extracellularly. The carboxyl‐terminal domain (CTD) of S1 and the amino‐terminal domain (NTD) of S6 are located in DI and DIV, respectively. The S4 subunit of each domain contains the voltage sensor. S5 and S6 of each repeat form the pore domain, and the connecting P‐loops between the S5 and S6 regions curve back into the pore to form the extracellular selectivity filter (SF), which is responsible for Na^+^ selectivity. ICL_III‐IV_ serves as an inactivation gate (IG), which closes the pore within 1‐2 ms after opening. The β subunit, consisting of an extracellular domain, an intracellular domain, and a single transmembrane helix, plays an important role in modulating the surface expression, kinetics, and functions of Nav channels. The β1 and β2 subunits do not stably associate with the Nav1.5 α subunit. Y304 in the Nav1.7 α subunit, which is connected to E48 in the β1subunit by a hydrogen bond, is substitute at L316 at the equivalent position in Nav1.5. Similarly, C895, which forms a disulfide bond with the β2 subunit, is substituted at L869. Several accessory proteins interact with Nav1.5 channels to form macromolecular complexes that regulate ion trafficking, posttranslational modifications and gating. Nav1.5 activity is driven by Ca^2+^ levels via its interactions with CaM and CaMKII. Nav1.5 can be activated in response to β adrenergic stimulation via the activation of CaMKII. CaM binds not only to an IQ motif in the CTD of Nav1.5 but also to its IG. The CaMKII inhibitor KN93 was recently reported to alter the kinetics of Nav1.5 inactivation by interrupting the CaM‐IG interaction but did not suppress CaMKII function.^64^

In ventricle cardiomyocytes, Nav1.5 channels are known to be located in lateral membrane, transverse tubules (T‐tubules), and intercalated discs, ensuring propagation of electrical impulse in longitudinal, transverse directions of cardiomyocytes, and between adjacent ones, respectively (Figure 1B).^49^ Nav1.5 channels are closed at the resting MP (Phase IV). In response to membrane depolarization, Nav1.5 could be activated. Within 200‐300 μs, a large inward peak I_Na_ (I_Na,P_) is formed to trigger overshooting of AP in Phase 0. At the end of this phase, most Nav1.5 channels are rapidly inactivated within 2‐5 ms, rendering the channel refractory until repolarization is completed in Phase III. During Phase IV, after recovering from inactivation, the channels are closed and can again be reopened by membrane depolarization (Figure 2A). In Phase II or III, a small population of total Nav1.5 channels could be reactivated before complete inactivation and then generate a relatively small, persistent sodium cardiac inward current called late I_Na_ (I_Na,L_).^50^, ^51^ Under physiological conditions, I_Na,P_ but not I_Na,L_ plays a central role in ventricular AP generation, while under pathological conditions, I_Na,L_ can play an important role.^50^, ^51^ Abnormal increases in I_Na,L_ prolong the duration of the AP plateau, triggering EADs or further elevating intracellular Ca^2+^ levels by driving the NCX exchanger to function in reverse mode, thereby inducing DADs and contributing to arrhythmogenesis.^51^

To date, some high‐resolution structures of Nav channels,^21^, ^52^, ^53^ including rNav1.5C,^18^ have been elucidated (Table 1). In general, key structural features of Nav1.5, the structural basis for its physiological function and its dysfunction in cardiac arrhythmias could be learned from the revealed Nav1.5 structures. Unlike other Nav α subunits, in Nav1.5, the regulatory interface with auxiliary β1 and β2 subunits, encoded by *SCN1B* and *SCN2B* respectively, is not as strong due to the substitution of residues for β subunit interactions.^18^ Recent studies suggest β1 subunit may differentially control expression and function of α‐subunit via acute and chronic feedback mechanisms.^54^ β2 is pivotal for the correct localization of NaV1.5.^55^ Nav1.5 is insensitive to the inhibition of tetrodotoxin (TTX), a selective sodium channel blocker nonprotein toxin, due to the substitution of binding residues at the outer mouth of the Nav1.5 selectivity filter (SF).^18^ Nav1.5 is blocked by the class Ic antiarrhythmic drug flecainide with comparatively high affinity and slow binding kinetics compared to class IA and IB antiarrhythmic drugs due to the larger hydrophobic ring structure of flecainide interacting with the central cavity of Nav1.5.^18^ In addition, the structural template of Nav1.5 for arrhythmia mutations provides a better understanding of the mechanism of various *SCN5A* variants in different positions.^18^ The traditional view is that a single α‐subunit of Nav1.5 functions as a monomer, while increasing evidence has shown that the α‐subunit of Nav1.5 could be oligomerized into dimers within the first intracellular loop and results in coupled gating properties with the accessory protein14‐3‐3 interaction.^56^ Inhibition of 14‐3‐3 could abolish the dominant negative (DN) effect and the biophysical coupling between α‐subunits.^56^

In addition, several accessory proteins have been demonstrated to interact directly with the α subunit of Nav1.5 channels (Figure 2B) to form macromolecular complexes with Nav1.5 and modulate the expression, trafficking and biophysical function of Nav1.5 (Table 2).^49^ Calmodulin (CaM), a ubiquitously expressed calcium‐binding protein, and CaM‐dependent protein kinase II (CaMKII), an adrenergically activated kinase, serve as important components affecting channel function.^34^ CaM binds with IQ motif of Nav1.5 carboxyl‐terminal domain (CTD) in Ca^2+^‐free forms and Ca^2+^‐bound forms at the basal levels of intracellular Ca^2+^ concentration.^33^, ^34^, ^35^ While this CaM‐Nav1.5 interaction is altered when the elevation of intracellular Ca^2+^ concentration, therefore changing the rate of Nav1.5 inactivation.^33^, ^34^, ^35^ Increasing evidence has shown that at the high level of intracellular Ca^2+^ concentration, CaM also directly binds to the inactivation gate (IG) of Nav1.5 to destabilize the IG and promote faster recovery from inactivation.^57^, ^58^, ^59^, ^60^, ^61^ CaMKII not only phosphorylates Nav1.5 at key site S571^62^ but also directly interacts with Nav1.5 to regulate the expression and function of Nav1.5.^63^ On the other hand, protein phosphatase 2A (PP2A) was recently found to interact with the Nav1.5/ankyrin‐G/CaMKII/Βiv‐spectrin macromolecular complex and balance CaMKII‐dependent phosphorylation.^62^ The CaMKII inhibitor KN93 but not autocamtide‐2‐related inhibitory peptide (AIP) could interrupt the CaM‐IG interaction by forming the ternary complex CaM‐IG‐KN93 and then inhibit Nav1.5 recovery from inactivation without altering the kinetics of inactivation.^64^ Therefore, determining the effects of accessory proteins and signaling pathways on modulating Nav1.5 provides us with a more comprehensive understanding of Nav1.5 roles in cardiac tissues in both health and disease states and is beneficial for the discovery of potential drug targets. Future investigations of the kinetics of CaM‐Nav complexes and the effects of structure‐guided mutations on the roles of Nav1.5 in the absence/presence of Ca^2+^ transients will provide us with a more comprehensive understanding of the mechanisms and significance of Ca^2+^‐dependent Nav roles in cardiac tissues in both healthy and disease states.

**TABLE 2:** Accessory proteins reported to interact with and regulate Nav1.5

Accessory proteins^239^, ^240^	Types^239^, ^240^	Binding sites	Biophysical function	Techniques
14‐3‐3η	Adaptor protein	ICL_I‐II_	Regulates Na^+^ current, negatively shifts the Nav1.5 inactivation curve and postpones recovery from inactivation without affecting the current density or Nav1.5 activation curve^241^	MPC
AP1‐ γ	Adaptor protein	Y1810 in the CTD	Forms a recognition site for Golgi to incorporate Nav1.5 into clathrin‐coated vesicles and transports to target membrane	MPC, immunostaining, mutagenesis
Ankyrin‐G	Anchoring‐adaptor protein	ICL_II‐III_	Regulates the accumulation of Nav1.5 on the membrane^63^, ^242^; E1053K eliminates ankyrin‐G binding, displays a faster onset of inactivation and slower recovery from inactivation, and negatively shifts channel activation curve^242^; 50% peak I_Na_ is reduced in Ankyrin‐G KO myocytes^63^	MPC, Co‐IP; immunostaining, mutagenesis
α‐actinin2	Cytoskeletal protein	ICL_III‐IV_	Anchors Nav1.5 to the actin cytoskeletalon network and increases sodium channel density without affecting gating properties^243^	MPC; Co‐IP
MOG1	Cofactor	ICL_II‐III_	Regulates the expression of Nav1.5, increases sodium current densities^244^	MPC, Co‐IP, WB
Synapse‐associated protein 97 (SAP97)	Anchoring‐adaptor protein	PDZ domain in CTD	Forms a complex with ankyrin‐G to regulate appropriate Nav1.5 expression, silencing SAP97 significantly reduces Na^+^ current^245^	MPC, WB, immunostaining
α1‐Syntrophin	Anchoring scaffold protein	PDZ domain in NTD and CTD	Enhances the expression of Nav1.5 and increases I_Na_ ^246^	MPC, Co‐IP, WB, immunostaining
Calmodulin (CaM), a calcium‐binding protein	Regulatory protein	IQ‐motif in CTD	Modulates slow inactivation thereby increasing the open probability of Nav1.5^247^	MPC, WB, immunostaining
		IG in ICL_III‐IV_	Destabilizes inactivation gate and promotes faster recovery from inactivation^57^, ^58^, ^59^, ^60^, ^61^; a CaMKII inhibitor KN93 interrupting the CaM‐IG interaction inhibits Nav1.5 recovery from inactivation without altering the kinetics of inactivation but doses not suppress the inhibitory function of CaMKII^64^	
CaM‐dependent protein kinase II (CaMKII)	Kinase	ICLI‐II	Forms a macromolecular complex with βIV‐spectrin and ankyrin‐G to regulate the expression and function of Nav1.5^62^, ^63^, ^248^; phosphorylates Nav1.5 at key site S571^62^, ^249^; serves as an important activator of I_Na,L_^62,^ ^250^	MPC, WB, immunostaining
Protein tyrosine phosphatase (PTPH1)	Enzyme	PDZ domain in CTD	Shifts the Nav1.5 hyperpolarized potentials^251^	MPC, WB
Caveolin‐3	Scaffolding and regulatory protein	Colocalize in the sarcolemma of human ventricle tissue^252^ Macromolecular complex including Nav1.5/α1‐Syntrophin/ nNOS/Caveolin‐3^81^	Serves as a negative regulator for cardiac I_Na,L_ via suppression of nNOS‐dependent direct S‐nitrosylation of Nav1.5^81^	MPC, Co‐IP, immunostaining

CTD, carboxyl terminal domain; NTD, N terminal domain; MPC, manual Qatch clamp; WB: Western blot; co‐IP, coimmunoprecipitation.

Mutations in *SCN5A* (Table **3**) are associated with inherited life‐threatening arrhythmias, such as long QT syndrome type 3 (LQTS3), and Brugada's syndrome (BrS).^18^, ^65^ Slower channel inactivation and thus conducting an increase in I_Na,L_ are responsible for the gain‐of‐function (GOF)‐associated LQTS3.^66^, ^67^ On the other side, reduction of membrane expression of functional channel due to synthesis deficiency^68^ or trafficking defects,^69^ impairment of gating (such as slower activation or faster inactivation)^56^, ^59^, ^70^, ^71^, ^72^ or permeation disruption^73^, ^74^ cause *SCN5A* loss‐of‐function (LOF)‐associated BrS.^65^ The α‐subunit of Nav1.5 oligomerization also explains the existence of several BrS variants displaying DN effects, providing new therapeutic targets for BrS caused by *SCN5A* LOF variants.^56^ Moreover, LOF mutations in *SCN1B* and *SCN2B* are also implicated in BrS.^54^, ^55^, ^75^, ^76^ In addition to most isolated GOF or LOF variants of *SCN5A* which are typically associated with a distinct clinical and electrocardiographic phenotype, variants could also lead to overlapping syndromes^77^, ^78^ or inherited arrhythmia syndrome different from BrS and LQT3.^79^ There is also a category of benign (atypical) *SCN5A* mutations which shows normal function alone but leads to a reduction in sodium currents when coexpressed with WT in vitro as typical *SCN5A* BrS mutations do.^80^

**TABLE 3:** Mutations in cardiac voltage‐gated Na^+^ channels subtypes associated with congenital syndromes

Subtypes	Encoding subunits gene	Congenital syndrome	Gain or loss of function	Mechanisms underlies the phenotype	Examples of variants
Nav1.1	*SCN1A*	Cardiac arrhythmia contributes to DS with SUDEP	LOF	Haploinsufficiency	R222X^235^: increases transient INa density, incidence of arrhythmogenic AP, EADs, DADs and rates of spontaneous contraction in DS patient iPSC‐CMs.
		SIDS	LOF	NR	G682V^236^: decreases sodium current in tsA201 cells expressing variant.
Nav1.4	*SCN4A*	Myotonia overlapping with BrS	NR	NR	V781I^237^
		Myotonia overlapping with prolonged QTc intervals	GOF	Gating defects	R1448C^46^: shows slower of inactivation and faster recovery time.
		SIDS	GOF	Gating defects	R1463S^238^: shows slower of inactivation and faster recovery time.
			LOF	Gating defects	V1442M^238^: shows enhancement of fast inactivation. E1520K^238^: shows reduction of current density.
Nav1.5	*SCN5A*	LQTS3	GOF	Gating defects	F1473C^66^: removes complete inactivation and thus conducting increase of I_Na,L_. A993T^67^: shows slower inactivation kinetics.
		BrS	LOF	Synthesis deficiency	W822X^68^: leads to the haploinsufficiency of the Nav1.5 protein and thus resulting in a nearly 50% reduction in Na^+^ current amplitude without significant alterations of biophysical properties and any dominant‐negative activity on wild type channels.
				Trafficking defects	D1690N^69^: produces a marked DN effect when cotransfected with wild‐type channels.
				Trafficking normal but gating defects	R878C^70^: nonconductive channel
					N1541D^71^: induces an accelerated entry into closed‐state inactivation.
				Gating defects	R1632C^71^, ^72^: produces an enhanced fast‐inactivated state stability because of a pronounced impairment of recovery from fast inactivation.
					R1629Q^56^: produces enhanced inactivation properties with a large hyperpolarizing shift in steady‐state inactivation, current densities similar to WT.
					A1924T^59^: reduces calmodulin binding and stabilizes Nav1.5 inactivation.
				Trafficking defects with gating defects	G1748D^69^: produces a marked DN effect, positively shifts the activation curve.
				Permeation disruption	E901K^77^, ^78^
		Benign (atypical) BrS	Normal function as WT	Normal	L567Q^80^: remains relatively unchanged current density, voltage‐current relationship, steady‐state inactivation, and recovery from inactivation, insufficient to produce a BrS phenotype. While, exerts DN effect on coexpression with WT via deficient trafficking mechanism.
		Overlap of BrS and LQTS3	Overlap GOF & LOF	Gating defects	E1784K^77^: exerts LOF effect via hyperpolarizing voltage dependence fast inactivation and accelerating rate of fast inactivation and GOF effect via destabilizing the IFM bound state of the channel to induce noninactivating currents.
		Different from LQT3 or BrS (In the absence of a distinct ECG phenotype)	Overlap GOF & LOF	Gating defects	C683R^79^: a novel variant, with the GOF effect resulting from a significant increase of the maximal current density and a hyperpolarizing shift of the steady‐state activation; without direct effect on I_Na,L_ at baseline or adrenergic stimulation, with the LOF effect resulting from an increased closed‐state inactivation.
	*SCN1B*	BrS	LOF	Gating defects	E87Q^75^: positively shifts the activation curve.
	*SCN2B*	BrS	LOF	Trafficking defects	D211G^76^: decreases Nav1.5 cell surface levels.
Nav1.6	*SCN8A*	Cardiac arrhythmia contributes to SUDEP in EIEE	GOF	No effect on *SCN8A* transcripts	N1768D^87^: increases calcium transient duration, prolongs the early phase of APD, and increases incidence of DADs but not changes in Nav1.5 expression.
Nav1.8	*SCN10A*	SUD	GOF	Gating defects	P1102S^89^: shows slower inactivation time course, allowing more Na^+^ to enter the cell.
		BrS	LOF	Gating defects	I671V^88^: depolarizes shift of activation and inactivation curve, reduces the sodium current.

NR, not reported; DS, Dravet syndrome; SUDEP, sudden unexpected death in epilepsy; SUD, sudden unexplained death; EIEE, early infantile epileptic encephalopathy; LQTS3, long QT syndrome type3; BrS, Brugada syndrome; GOF, SIDS, sudden infant death syndrome; gain‐of‐function; LOF, loss‐of‐function; DN, dominant negative.

Moreover, missense variants in *CAV3*‐encoded caveolin‐3, which forms macromolecules with Nav1.5 and serves as a negative regulator for I_Na,L_, could result in I_Na,L_ increase and thus cause LQTS9, providing new therapeutic strategies to correct I_Na,L_.^81^ Drugs that inhibit I_Na,L_^50^ could shorten the AP duration or QT interval and could therefore be considered a potential treatments for I_Na,L_‐associated diseases.^82^ Thus, abnormal changes in I_Na,L_ could be considered as a target for drug development and safety evaluation.

High‐throughput assays of cardiac Nav1.5 I_Na,P_ have been widely used in cardiac safety screening, but screening studies do not routinely measure I_Na,L_.^83^ However, it is important for potential therapeutic candidates that could minimize I_Na,L_ without affecting I_Na,P_ to be selected.^51^ The variety of different protocols and measurement strategies applied in the use of these drugs have contributed to remarkable variations in the reported data on I_Na,L_ and screening results for inhibitory compounds.^84^, ^85^ I_Na,L_ is small, and studies have had difficulty generating reproducible data; thus, the best choice for an I_Na,L_ enhancer should increase I_Na,L_ with no obvious effect on I_Na,P_.^85^ In addition, it is necessary to double check the median inhibitory concentration (IC_50_) of potential drugs in the absence of enhancers, eliminating the modification effect of enhancers on the activity of compounds,^85^ and to evaluate the IC_50_ of drugs in different stimulation states with regard to variations in the effects on I_Na,P_ and I_Na,L_ in different stimulation states.^18^

#### 4.1.2 Other Nav channels in the heart

TTX‐sensitive Nav channels including neuronal Nav (eg, *SCN1A*‐encoding Nav1.1, *SCN2A*‐encoding Nav1.2, *SCN3A*‐encoding Nav1.3, *SCN8A*‐encoding Nav1.6), which were first identified in neurons, and skeletal muscle Nav (eg, *SCN4A*‐encoding Nav1.4), which was first identified in skeletal muscle, have been unexpectedly found in T‐tubules of ventricle myocytes (Table 1), contributing a small portion to the total sodium current under physiological conditions due to their much lower expression level than Nav1.5.^39^, ^40^, ^41^, ^45^, ^46^, ^47^ While, in inherited forms of cardiac arrhythmia, augmentation of TTX‐sensitive Nav channels phosphorylated by β‐AR stimulation/CaMKII stimulation, contributes to abnormal increases in I_Na,L_ and arrhythmogenic Ca^2+^ release.^32^, ^41^ Compared with other TTX‐sensitive Nav channels, the location of Nav1.6 (Table 1) is the closest channel to RyR2 (< 100 nm)^41^, ^86^ indicating that Nav1.6 is capable of impacting Ca^2+^ cycling proteins and Ca^2+^ dynamics in both health and disease.^86^ GOF variants of *SCN8A*‐encoding Nav1.6 (Table 3) potentially leads to sudden unexpected death in epilepsy (SUDEP) due to arrhythmia of the brain and the heart.^87^ The GOF variant of *SCN8A* (N1768D) causes hyperexcitability of ventricle myocytes by increasing calcium transient duration, prolonging the early phase of APD, and increasing the incidence of DADs but not by compensatory changes in Nav1.5 expression.^87^ Selective pharmacological blockade of Nav1.6 and silencing of Nav1.6 indicate that Nav1.6 can potentially contribute to β‐AR stimulation‐induced I_Na,L_ and arrhythmias.^41^ This explains why catecholaminergic polymorphic ventricular tachycardia (CPVT) models respond to treatment with some Na^+^ channel blockers.^41^ Besides *SCN8A*, the possible roles of *SCN1A*, *or SCN4A* mutations in pathophysiology of cardiac congenital syndrome were also investigated (Table 3).

In addition, TTX‐insensitive *SCN10A*‐encoding Nav1.8 (Table 1) channels, which are mainly expressed in the peripheral nervous system, are also found in the heart^42^, ^43^, ^44^, ^48^ at a higher level in the atrial myocardium than in the ventricular myocardium,^43^ exhibiting a more depolarized voltage dependence of inactivation and slower inactivation kinetics than other faster sodium channels like Nav1.5.^44^ Nav1.8 contributes to abnormal increases in I_Na,L_ and consequently prolongs the APD and elevates proarrhythmogenic diastolic SR Ca^2+^ in cardiac disease.^42^ Genetic deletion of Nav1.8 produces a smaller I_Na,L_ increase than in WT cardiomyocytes during β‐AR stimulation.^42^ LOF and GOF variants in *SCN10A* (Table 3) are associated with BrS^88^ and SUDEP,^89^ respectively. Gating dysfunction with enhanced of inactivation results in LOF of Nav1.8.^88^ In contrast, dysfunction with slower inactivation could result in GOF of Nav1.8 and then allow more Na current entry.^89^ Thus, Nav1.8 also plays a significant role in the initiation of proarrhythmic triggers via I_Na,L_‐induced SR Ca^2+^ leakage.

### 4.2 Ca channels

In response to membrane depolarization, voltage‐gated calcium (Cav) channels activate and mediate extracellular Ca^2+^ influx into the cytosol, which serves as the second messenger of electrical signaling, initiating many physiological processes, such as excitability, contraction and cell death.^90^ The Cav1 and Cav3 groups mediate L‐type and T‐type currents, respectively, and are involved in cardiac function. Cav1 is more highly expressed than Cav3 in ventricular myocytes, while Cav3 is mainly expressed in SAN cells (Table 4).^90^ Ryanodine receptors (RyRs), intracellular Ca^2+^ channels in the sarcoplasmic/endoplasmic reticulum (SR/ER), control the rapid release of Ca^2+^ from SR/ER into the cytoplasm to initiate CICR, a key event that triggers skeletal and cardiac muscle contraction.^91^, ^92^ Among three mammalian isoforms (RyR1, RyR2, and RyR3), RyR2 is primarily expressed in cardiac muscles, ^91^, ^92^, ^93^ and higher expressed in the ventricle (Table 4).^37^, ^38^

**TABLE 4:** Cardiac Ca^2+^ channels subtypes

Cav types	Subtypes	Encoding α subunits gene	Auxiliary subunits	Main location	Subcellular localizationin cardiac tissue (V/A/SAN)^37^, ^38^ and region	Cryo‐EM Structure	Principal physiological functions in human ventricle myocytes
L (long lasting and large conductance) Type	Cav1.2	*CACNA1C*	α2δ and intracellular β, KchIP2	Heart, CNS	V≈A > SAN T‐tubule^90^	NR, refers to Cav1.1	Mediates the entry of Ca2+, contributes the AP plateau and initiates excitation‐contraction coupling in cardiac muscle
	Cav1.3	*CACNA1D*	NR	CNS, Heart	V< A < SAN	NR	Cardiac SAN pacemaker activity
T (transient‐opening and small conductance) Type	Cav3.1	*CACNA1G*	α2δ	Heart, CNS	V< A < SAN	Human Cav3.1 complex containing α1, α2δ subunits^253^; providing the structural reason why less energy is required for Cav3.1 to open the intracellular gate and facilitating the activation at lower voltages^253^	Cardiac SAN pacemaker activity
	Cav3.2	*CACNA1H*	NR	Heart, CNS	V≈A < SAN	NR	Cardiac SAN pacemaker activity
Intracellular calcium channel	RYR2	*RYR2*	NA	Heart	V≈A ≫SAN	Porcine Ryr2^110^	Controls rapid release of Ca^2+^ from SR/ER into cytoplasm to initiate CICR

CNS, central nervous system; V, ventricle; A, atrium; SAN, sinoatrial node; Cryo‐EM, cryoelectron microscopy; NA: not available; NR, not reported.

#### 4.2.1 Cav1.2

**FIGURE 3:**
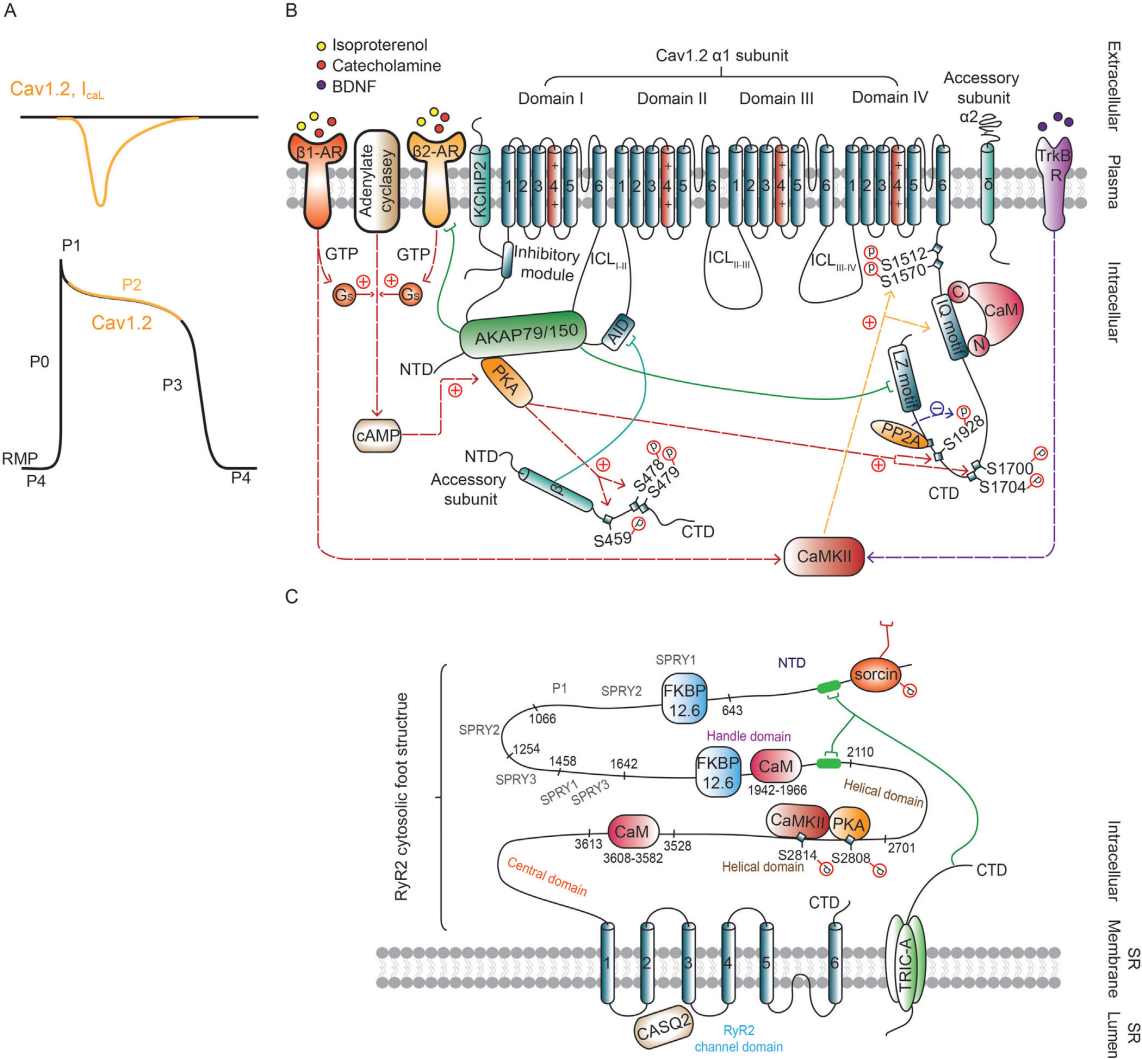
Cardiac voltage‐gated L‐type calcium channel (Cav1.2) and Ryr2 structure, accessory proteins and signaling. (A) The contribution of inward current I_CaL_ (upper) mediated by Cav1.2 to action potential (AP) Phase II (lower). (B) The L‐type calcium channel Cav1.2 is formed by the interaction of the pore‐forming α1 core subunit with auxiliary subunits, including α2δ and intracellular β. The α1 subunit consists of four homologous domains with a voltage sensor S4 and pore‐forming S5 and S6 in each domain and connected by cytoplasmic linkers (ICL_I‐II_, ICL_II‐III_, and ICL_III‐IV_). The β subunit is localized exclusively at the cytosolic face of the channel and its GK domain interacts with the α‐interaction domain (AID) of the α1 subunit I‐II loop to mediate Cav1.2 trafficking by antagonizing ER retention signals. The α2δ subunit binds to extracellular regions, including domain III of the α1c subunit. δ is linked with a larger α2 polypeptide via a disulfide bridge. The α1 subunit interacts with several proteins, receptors and subunits of other channels. The amino‐terminal domain NTD, ICL_I‐II_ and carboxyl‐terminal domain (CTD; LZ motif) of Cav1.2 interact with AKAP. PKA and β2‐AR CTD also bind to AKAP. PP2A binds to site next to S1928 in the CTD. CaM binds to the IQ motif. KChIP2, an accessory subunit of Kv4.3, directly interacts with the inhibitory module at the NTD of the Cav1.2 α1 subunit. The Cav1.2 β subunit interacts with the AID of the α1 subunit I‐II loop. The β‐AR/cAMP/PKA, β1‐AR/CaMKII, TrkB R/CaMKII signaling pathways are involved in modulating the expression and function of Cav1.2 in the heart. PKA‐related phosphorylation pathway (red arrows) and CaMKII‐related phosphorylation pathway (purple arrows). (C) RyR2 is a large, square, homotetramer in the configuration of a four‐leaf clover. Each subunit of the homotetramer consists of a large cytosolic domain (called the foot structure), which is responsible for interaction with protein modulators, and CTTD. Four identical carboxyl‐terminal transmembrane domains (CTTDs) are responsible for forming the central ion‐conducting pore. RyR2 is located beneath Cav1.2 in T‐tubules and is connected with α1 subunit of Cav1.2 by sorcin. In addition, CaM and FKBP12/12.6 also interact with the cytosolic foot structure. Kinase (PKA, CaMKII) and phosphatase (PP1 and PP2A) exert phosphorylation/dephosphorylation effects on Ryr2. Type 2 calsequestrin (CASQ2) interacts with the luminal surface of Ryr2 to increase the open probability. In addition, class A of trimeric intracellular cation (TRIC‐A) channels on the SR membrane directly interact with the cytosolic region of RyR2 (Figure 1B) via its carboxyl‐terminal tail domains to facilitate Ca^2+^ release from the SR

Cav1.2 channels, located in T‐tubules of ventricular myocytes (Figure 1A), are assumed to be the major subtype of Cav1 channels that mediate the entry of Ca^2+^, which is required for the AP plateau (Figure 3A), and EC coupling, triggering activation of RyR2 and initiating CICR (Figure 1A).^90^

The Cav1.2 complex consists of one pore‐forming subunit α1c and the auxilary subunits α2δ and intracellular β (Figure 3B).^94^, ^95^, ^96^, ^97^, ^98^ The full‐length cryo‐EM structure of cardiac Cav1.2 has not been revealed, while skeletal Cav1.1 was the first Cav channel to have its full‐length cryo‐EM structure reported with an overall resolution of 4.2 and 3.6 Å.^99^, ^100^ The structure of Cav1.1 provides a structural template for the Cav1 family and comparisons for molecular interpretations of the functions and disease mechanisms between eukaryotic Cav and Nav channels.^99^, ^100^ Because the α1 subunits from Cav1.1 and Cav1.2 are highly homologous, Cav1.2 modeling could be based on the Cav1.1 structure for analyzing the molecular determinants of opening and closure of Cav1.2.^94^ Voltage‐independent upward movement or voltage‐dependent movement of S4 segments maintain the opening or closure of the gating, respectively.^94^ The voltage sensor S4‐S5 are coupled with pore S6 segments by directly interacting with a ring of small residues, which are regarded as interesting sites for studying electromechanical coupling.^94^ In addition, in complex with channel‐selective agonists/antagonists, structural analysis helps to elucidate their specific binding sites and reveal the structural reasons why similar types of molecules (such as nifedipine and Bay K8644) exert opposite antagonist and agonist effects on Cav1.1 channels.^20^ The auxiliary subunits α2δ and β generally modulate the surface expression and biophysical kinetics of α1c.^97^, ^98^ Recent studies have raised a new function for β subunits in hearts: β subunit binding to α1c might be dispensable for Cav1.2 trafficking at normal physiological conditions but is essential for the augmentation of Ca^2+^ current and cardiac contractile response to β‐adrenergic stimulation.^96^

In addition, several accessory proteins interact with the α1c subunit of Cav1.2 and regulate Cav1.2 expression and function (Table 6). Cav1.2 is involved in the β‐AR/cAMP/PKA signaling pathway^95^ (Figure 3B). β1‐AR/cAMP signaling is diffusive and global, while β2‐AR/cAMP is relatively localized.^95^ The CTD of β2‐AR not only binds to A‐kinase anchoring protein (AKAP) but also directly binds to Cav1.2 to mediate local signaling via the cAMP‐dependent PKA pathway and facilitate localized cAMP signaling.^95^ The PKA‐dependent phosphorylation of amino acids in the CTD of the α1c subunit has been demonstrated to be decisive for the β‐AR‐mediated upregulation of cardiac I_CaL_.^95^, ^101^ In addition, some amino acids in the CTD of the α1c subunit are targets of the β1‐AR/CaMKII signaling pathway.^95^ In parallel with the roles of the β‐AR system, BDNF‐TrkB binding regulates myocardial Ca^2+^ cycling and EC coupling by triggering CaMKII‐dependent signaling.^102^

GOF variants in *CACAN1C* (Table 5) cause timothy syndrome (TS), which is a multisystemic disorder including LQTS8, autism, and dysmorphic features.^103^ Complete loss of inactivation kinetics leading to a prolonged calcium influx during action potentials,^103^ or left shift in the activation curve leading to increase in window currents^104^ could result in GOF of the Cav1.2 channel. Variant E1496K slowed inactivation, causing isolated LQTS8 without TS.^105^ On the other side, LOF variants (Table 5) which disruption of protein trafficking,^106^, ^107^ gating,^108^ or Ca^2+^ permeation^108^, ^109^ account for genotyped BrS cases. These results implicated the importance of the Cav1.2‐mediated calcium signaling in human physiology and heart disease.

**TABLE 5:** Mutations in cardiac Ca^2+^ channels subtypes associated with congenital syndromes

Subtypes	Encoding subunits gene	Congenital syndrome	Gain or Loss of function	Mechanisms underlies the phenotype	Examples of variants
Cav1.2	*CACNA1C*	TS	GOF	Gating dysfunction	G406R^103^: leads to a prolonged calcium influx during action potentials caused by complete loss of voltage‐dependent channel inactivation.
					G419R^104^: displays a 4‐fold increase in the peak current density and a left shift in the activation curve resulting in increase in window currents.
		Isolated LQT8 without causing TS	GOF	Gating dysfunction	E1496K^105^: slows inactivation and thus might contribute to prolonged action potential duration.
		BrS3	LOF	Trafficking defects	A39V,^106^T320M/Q428E^107^
				Gating defects	V2014I^108^: significantly reduces conductance of the calcium channel at potentials between 0 and +30 mV during activation, shifts half‐inactivation voltage to more negative potentials.
				Permeation disruption	E1115K^108^, ^109^: destroys the calcium selectivity, and instead converts the mutant channel into a channel with a marked increase in sodium‐mediated inward currents and potassium‐mediated outward currents.
Cav1.3	*CACNA1D*	SANDD	LOF	Gating defects	403_404insGly^254^
Cav3.1	*CACNA1G*	Bradycardia, atrioventricular conduction block	LOF	NR	NR
RYR2	*RYR2*	CPVT	GOF	Gating defects	R176Q^125^: increases probability of channel opening, increases incidence of spontaneous Ca^2+^ oscillations thus causing susceptibility to CPVT.
				Channel instability	S2246L^124^: disrupts the interdomain interactions after channel activation and increases channel opening.
		CRDS which could cause SCD without CPVT	LOF	Gating defects	D4646A^126^: impairs the cytosolic Ca^2+^ activation and diminishes the luminal Ca^2+^ activation of single RyR2 channels; suppresses catecholamine‐induced SR Ca^2+^ release and produces systolic arrhythmogenic abnormalities without affecting expression.

TS, Timothy syndrome; SANDD, sinoatrial node dysfunction and deafness syndrome; LQTS, long QT syndrome; CPVT, catecholamine‐induced ventricular arrhythmias; SCD, sudden cardiac death; CICR, Ca^2+^‐induced Ca^2+^‐release; CRDS, Ca^2+^ release deficiency syndrome; GOF, gain‐of‐function; LOF, loss‐of‐ function.

#### 4.2.2 Ryr2

The near‐atomic‐resolution cryo‐EM structure of RyR2 from porcine hearts has been recently revealed in both the open and closed states,^110^ or with key modulators,^111^, ^112^ offering the opportunity to characterize the roles of the structural elements and modulators during gating shifts. Each subunit of the homotetrameric RyR2 consists of a large cytosolic domain, which is responsible for interaction with protein modulators, linking gap between the SR and transverse tubule (T‐tubule) membranes, and carboxyl‐terminal transmembrane domain (CTTD), four identical of which are responsible for forming the central ion‐conducting pore (Figure 3C).^110^ The cryo‐EM structures of the RyR2 complex and the abovementioned Cav1.1 establish a solid foundation for future revealing the Cav1.2 complex, the complex formation between Cav1.2 and RyR2, and excitation‐contraction coupling.

Several proteins (Table 6) interact with the cytosolic region of Ryr2 to regulate its open probability. For example, CaM^91^ inactivates Ryr2 during diastolic cytosolic calcium elevation, thus playing an important role in Ca^2+^ alternans.^113^ The CaM binding sites on cytosolic sites of Ryr2 will be shifted and dependent on Ca^2+^ concentration binding to CaM.^112^ Enhancement of CaM function promotes, whereas impairment of CaM function suppresses Ca^2+^ alternans.^113^ Several enzymes, such as PKA, CaMKII, PP1, and PP2A, interact with Ryr2 and exert phosphorylation/dephosphorylation effects on Ryr2.^114^ The hyperphosphorylation of RyR2 by PKA^114^ and/or by CaMKII^115^ causes abnormal Ca^2+^ leakage from the SR. RyR2 is also coupled to proteins at the luminal SR surface, such as type 2 calsequestrin (CASQ2),^116^, ^117^ which increases the open probability and facilitates high rates of Ca^2+^ efflux during systole.^116^

**TABLE 6:** Accessory proteins reported to interact with and regulate Cav1.2 and RYR2

Cav1.2				
Accessory proteins	Types	Binding sites	Biophysical function	Techniques
Bridging integrator 1 (BIN1)	Scaffolding protein	Adjacent to Cav1.2 channels clustered in T‐tubules	BIN1 is responsible for Cav1.2 trafficking to T‐tubules; knockdown of BIN1 decreases the surface expression of Cav1.2 and calcium transients in mouse cardiomyocytes^255^, ^256^; BIN1 increases Cav1.2 channel clustering and whole‐cell Ca^2+^ currents in human embryonic stem cell‐derived cardiomyocyte (hESC‐CM) ^257^	MPC, Ca^2+^ imaging, WB
AKAP 79/150	Anchoring protein	In the NTD, ICL_I‐II,_ and LZ motif in the CTD of α1 subunit	Forms macromolecular complex with Cav1.2 and takes part in different regulatory pathways by recruiting several signaling molecules, such as PKA to Cav1.2^95^; PKA‐AKPA interaction is disrupted by the membrane‐permeable stearylated peptide Ht31^95^	WB
KChIP2	Accessory subunit of Kv4.3	In the NTD of the α1 subunit^258^	Modulates the Cav1.2 current without affecting Cav1.2 protein expression or trafficking^258^	MPC, WB
CaM	Regulatory protein	IQ motif in the CTD^259^	Facilitates the Cav1.2‐Cav1.2 channel interactions within a cluster and then work cooperatively^259^	MPC, Ca^2+^ imaging
PKA	Kinase	Recruited via AKAP	Upregulation of L‐type currents by phosphorylates S1700/T1704,^101^ S1928^95^ in the CTD	MPC
PP2A	Phosphatase	Between S1928 and LZ motif in the CTD	Antagonizes β‐AR/PKA mediates phosphorylation of Cav1.2 and upregulation of L‐type currents^260^, ^261^	MPC

Ryr2				
Accessory proteins	Types	Binding sites	Biophysical function	Techniques
FK506 binding proteins (FKBP12/12.6)	Regulatory protein	Cytosolic region	Stabilizes RyR2 in the closed state, reduces its activity, prevent aberrant activation of the channel during the resting phase of the cardiac cycle^262^	Single‐channel recordings, MPC
Sorcin	Calcium binding protein	cytosolic region; CTD of the α1 subunits of Cav1.2	Sorcin completely inhibits ryanodine binding to cardiac RyRs, reduces the open probability of Ryr2^263^ and Cav1.2, and bridges the gap between SR and the sarcolemma for interchannel cross‐talk^118^	WB, Co‐IP, single‐channel recordings
CaM	Regulatory protein	Cytosolic region	Inactivate Ryr2 during diastolic cytosolic calcium elevation, thus playing an important role in Ca2^+^ alternans^113^	Ca^2+^ imaging, MPC
CaMKII	Kinase	Cytosolic region	Phosphorylates of RyR2, regulates the channel open probability^115^	Single‐channel recordings, Ca^2+^ imaging
PKA	Kinase	Cytosolic region, S2809	Phosphorylates of RyR2 and dissociates FKBP12.6, regulates the channel open probability^114^	Single‐channel recordings, WB
TRIC‐A	Regulatory protein	Cytosolic region	Serves as counterion channels that provide the flow of K^+^ ions into the SR during the acute phase of Ca^2+^ release and thereby facilitates Ca^2+^ release from the SR^8^, ^121^	Single‐channel recordings
Type 2 calsequestrin (CASQ2)	Regulatory protein	Luminal region	Increases the open probability of RyR2^116^	Single‐channel recordings, WB

CTD, carboxyl terminal domain; NTD, N terminal domain; MPC, manual Qatch clamp; WB: Western blot; co‐IP, coimmunoprecipitation.

Moreover, RyR2 also interact with other channels. RyR2 is located beneath most Cav1.2 (within ∼12 nm) in T‐tubules and is connected with the α1 subunit of Cav1.2 by sorcin, which is a Ca^2+^‐binding protein reducing the open probability of Ryr2, bridging the gap between SR and the sarcolemma for interchannel cross‐talk.^118^ In addition, trimeric intracellular cation (TRIC) channels represent a recently discovered class of cation channels that were first identified in rabbit skeletal muscle in 2007.^119^ TRIC‐A is a subtype that is abundantly expressed in excitable cells, having slightly higher permeability for K^+^ than Na^+^ and mediating counterion movements by releasing Ca^2+^ from the SR.^120^ The cryo‐EM structure of the symmetrical trimer TRIC‐A has been reported.^8^ Moreover, TRIC‐A also directly interacts with the cytosolic region of RyR2 via its carboxyl‐terminal tail domains (Figure 3C) to modulate intracellular Ca^2+^ homeostasis and thereby facilitates Ca^2+^ release from the SR.^121^ The open probability and current amplitude of TRIC‐A are increased by a positive shift in the MP^8^ but are blocked by exposure to a high‐concentration Ca^2+^ bath on the luminal side during the resting state.^8^, ^121^ TRIC‐A gene deletion decreases the sensitivity of individual RyR channels to β‐AR/PKA stimulation, eventually resulting in Ca^2+^ release impairment^122^ and irregular ECG.^121^ These studies indicate that TRIC‐A promotes the release of Ca^2+^ from the SR via RyR2 and maintains RyR2 function at low Ca^2+^ to neutralize the transient luminal negative charge caused by Ca^2+^ release in cardiomyocytes.

GOF variants in RyR2 (Table 5) are implicated in ventricular tachyarrhythmias, including type 1 of CPVT type (CPVT1), which is characterized by stress‐induced ventricular tachycardia in the absence of a structurally abnormal heart.^123^ GOF variants could induce channel instability by disrupting the interdomain interactions after channel activation,^124^ or increase the open probability of RyR2 and pathological SR Ca^2+^ release,^115^, ^125^ and thus causing susceptibility to CPVT. On the other hand, RyR2 LOF variants have been identified among survivors of cardiac arrest without exhibiting the CPVT phenotype and further regarded as RyR2 Ca^2+^ release deficiency syndrome (CRDS) via an EAD‐mediated mechanism.^126^ I_to_, I_CaL_, and I_NCX_ were alternatively increased, although catecholamine‐induced SR Ca^2+^ release was suppressed in LOF variant D4646A, thus causing AP waveform alteration and finally enhancing the propensity for arrhythmogenic EADs.^126^

In CPVT cardiomyocytes with the RyR2 variant R176Q, a viral vector containing a CaMKII inhibitor (autocamtide‐2‐related inhibitory peptide, AAV9‐GFP‐AIP) completely suppressed the abnormal increase in spontaneous Ca^2+^ transients, suggesting that CaMKII suppression represents a potential therapy for CPVT.^127^ A KN93‐mediated increase in RyR2 Ca^2+^ release in cardiomyocytes was found to be due to disruption of the CaM‐RyR2 interaction rather than inhibition of CaMKII.^64^ Gene transfer of CaM, exhibiting a slower Ca^2+^ dissociation rate and longer RyR2 refractoriness, alleviated arrhythmias in a CASQ2‐associated CPVT mouse model.^128^ Previous studies have illustrated that flecainide prevents ventricular tachyarrhythmia in patients with CPVT by blocking of the TTX‐sensitive Nav channel.^41^ Recent research has shown that the antiarrhythmic effect of flecainide mainly relies on blocking RyR2 channels but not TTX‐sensitive Nav channels.^129^ The secondary amine on the piperidine ring in flecainide is necessary for its activity in RyR2 channels.^129^ In general, the regulation of RyR2 modulators (RyR2‐CaM interaction) represents an important therapeutic target for regulating cardiac alternans in cardiac ventricular arrhythmia.

### 4.3 Kv channels

Cardiac Kv channels play prominent roles in resting potential maintenance, AP repolarization, and the AP plateau phase.^130^, ^131^ For example, Kv1.4/Kv4.3, Kir2.1, Kv11.1, and Kv7.1 are highly expressed in the ventricular myocytes (Table 7).^37^, ^38^ Kir2.1 contributes to the maintenance of the resting potential in Phase IV, while Kv4.3 and Kv1.4 contribute to repolarization, specifically the notch (the transient repolarization period) of the AP^130^, ^131^. Of particular relevance to the AP plateau is the delayed rectifier current (I_K_), which includes rapid (I_Kr_) and slow (I_Ks_) components that are governed by distinct channel subtypes Kv11.1 and Kv7.1, respectively.^130^, ^131^ Dysfunction of cardiac Kv channels can result in APD changes and the subsequent development of LQTS, SQTS, or other related life‐threatening ventricular arrhythmias or sudden cardiac death.^1^, ^132^

**TABLE 7:** Cardiac voltage‐gated K^+^ channels

Potassium channel types	Subtypes	α Subunits sene	Auxiliary subunits	Main location	Subcellular localization in cardiac tissue (V/A/SAN)^37^, ^38^, ^264^ and region	Cryo‐EM structure	Principal physiological functions in human ventricle myocytes
Voltage‐dependent K^+^ channels 1‐9, Shaker‐related channels, containing six transmembrane regions (S1‐S6) with a single pore	Kv1.4	*KCNA4*	Kvβ1.2	Heart	V >A≈SAN T‐tubules, IDs	NR	Mediates I_to, slow_ and contributes to early AP repolarization
	Kv4.3	*KCND3*	KChIP2 encoded by *KCNIP2*; Navβ1 encoded by *SCN1B*	CNS, Heart	V< A > SAN; T‐tubules	NR	Mediates I_to, fast_ and contributes to early ventricular AP repolarization
	Kv7.1	*KCNQ1*	MinK encoded *by KCNE1*	Heart	V> A > SAN; IDs, lateral membrane, T‐tubules	Frog KCNQ‐CaM complex in an uncoupled, PIP2‐free state^144^	Mediates I_ks_ and contributes to Phase II,III AP repolarization and early Phase IV of the AP
Voltage‐dependent K^+^ channels 10‐12, nonshaker‐related channels	Kv11.1	*KCNH2*	MinK and MiRP encoded by *KCNE1* and *KCNE2*, repectively	Heart, CNS	V≈A > SAN IDs, lateral membrane, T‐tubules	Human Kv11.1^19^	Mediates I_ks_ and contributes to Phase II, III AP repolarization and early Phase IV of the AP
Inward rectifying K^+^ current, containing only two trans‐ membrane regions and a single pore	Kir2.1	*KCNJ2*	NA	CNS, Heart	V>A≫SAN; T‐tubules	NR	Mediates I_k1_ and contributes to Phase IV resting MP and the terminal Phase III repolarization

CNS, central nervous system; IDs, intercalated discs; V, ventricle; A, atrium; SAN, sinoatrial node; Cryo‐EM, cryoelectron microscopy; KChIP2, K+ channel interacting protein 2; NA: not available; NR, not reported.

#### 4.3.1 Kv4.3

**FIGURE 4:**
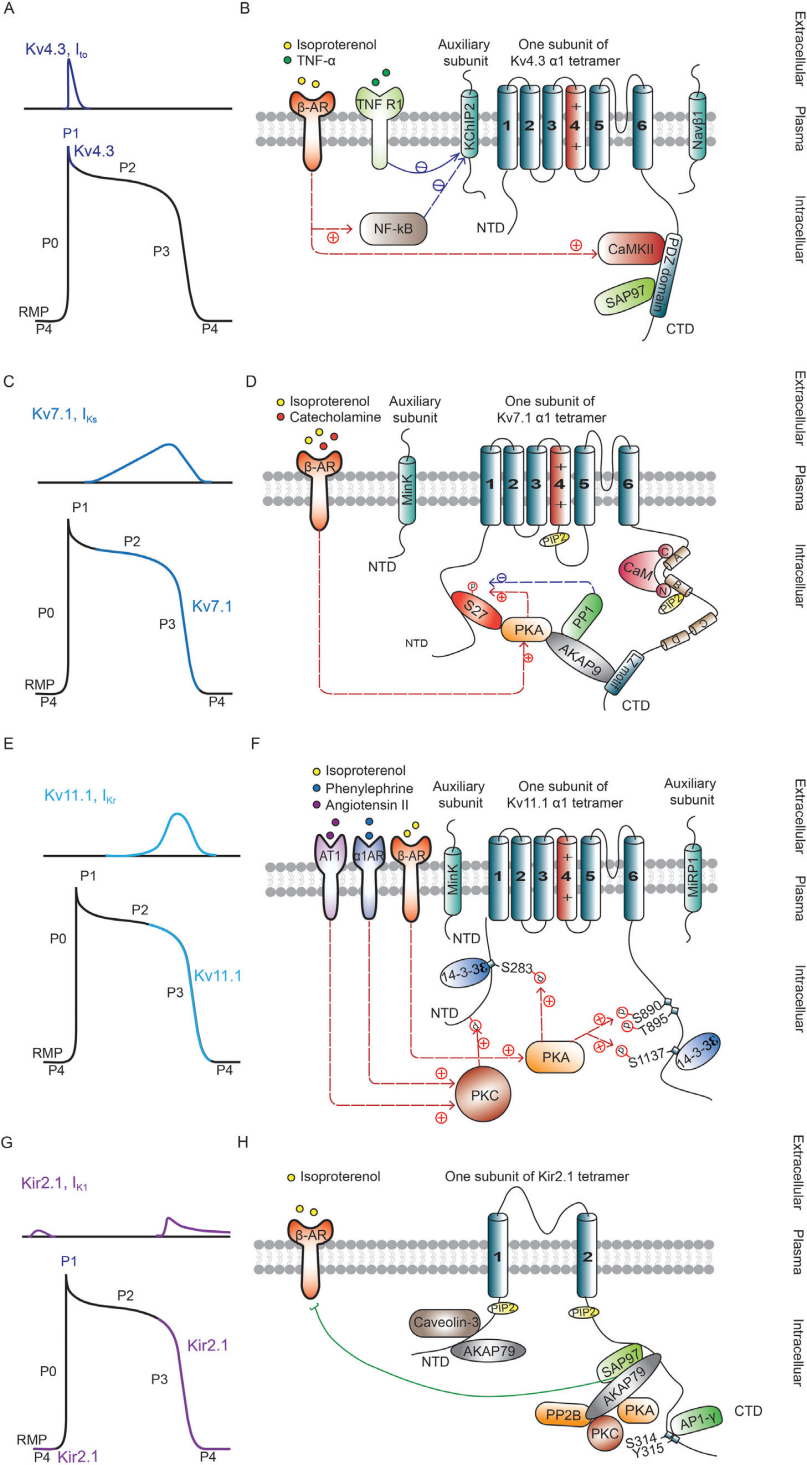
Cardiac voltage‐gated potassium channel structure, accessory proteins and signaling. (A) The contribution of the outward current I_to_ (upper) mediated by Kv4.3 to action potential (AP) Phase I (lower). (B) Kv4.3 is formed by the α subunit and accessory protein K^+^ channel interacting protein 2 (KChIP2, β subunit). A tripartite complex including the anchoring protein SAP97 and kinase CaMKII is formed at the Kv4.3 carboxyl‐terminal domain (CTD) via a PDZ domain‐binding motif Ser‐Ala‐Leu (SAL). The channel current is modulated by CaMKII and NF‐kB activation, which are themselves mediated by β‐AR stimulation. (C) The contribution of the outward current I_Ks_ (upper) is mediated by the delayed rectifier potassium channel (Kv7.1) to AP Phase II and III (lower). (D) Kv7.1 is formed from the α1 subunit, which consists of four homologous domains with avoltage sensing S4 segment and pore‐forming S5 and S6 segments in each domain. Auxiliary subunit *KCNE1* interacts with residue V141 of S1 in *KCNQ1* to allow the complex only open from a fully activated S4 conformation by altering the VSD S4‐to‐gate coupling, and also interacts with F339 in KCNQ1 to reduce the open probability at negative voltages. PIP2 binds to the S4‐S5 linker during membrane depolarization. CaM binds at the Kv7.1 C‐terminus, where it can compete with and replace PIP2. CaM also interacts with the Kv7.1 voltage sensor domain. Kv7.1 is modulated by the β‐AR/PKA pathway, which phosphorylates Kv7.1 at its amino‐terminal domain (NTD; S27). A‐kinase anchoring protein 9 (AKAP9) interacts with the LZ motif in the Kv7.1 CTD and is anchored by PKA and PP1. (E) The contribution of the outward current I_Kr_ (upper) mediated by the inwardly rectifying potassium channel (Kv11.1) to AP Phase II and III (lower). (F) The α subunit of Kv11.1 contains six transmembrane helices, with S4 acting as the voltage sensor and S5‐S6 forming the pore. The KCNE β subunits KCNE1 and KCNE2 interact with Kv11.1. The adaptor protein 14‐3‐3ℇ interacts with Kv11.1 in the NTD and CTD. β1‐AR competes with Kv11.1 for association with 14‐3‐3ℇ proteins. β‐AR/PKA, α1‐AR/PKC, and AT1/PKC are involved in Kv11.1 channel modulation. PKA and PKC phosphorylate the α1 subunit (red arrows). (G) The contribution of the strong inward rectifier potassium current I_K1_ (upper) mediated by the Kir2.1 channel to AP Phase 0, III, and IV (lower). (H) Kir2.1 channels have two membrane‐spanning domains; a p‐loop that forms the ion selectivity filter and intracellular N‐ and C‐terminal domains. The CTD of Kir2.1 directly associates with AP1, AKAP79, and SAP97. SAP97 also interacts with β1‐AR and to modulates the effect of β1‐AR on Kir2.1. AKAP79 can bind with SAP97 and also anchor kinases (PKA, PKC), and phosphatase (PP2B) close to Kir2.1phosphorylation sites. The NTD of Kir2.1 directly interact with, caveolin‐3 which regulates Kir2.1 trafficking and surface expression. PIP_2_ binds to both the CTD and the NTD to modulate channel gating

The rapidly activated and inactivated transient outward potassium current (I_to_) contributes to early ventricular AP repolarization and underlies the initial “notch” before the AP plateau phase in humans and other larger mammals (Figure 4A).^130^ I_to, fast_ and I_to, slow_ are the two distinct components of I_to_, and are mediated by Kv4.3 and Kv1.4, respectively, in humans and by Kv4.2/Kv4.3 and Kv1.4, respectively, in rodents.^1^ Unlike in human and mammalian models, ventricular AP in rodent models exhibits fast repolarization without a plateau phase due to I_to_ rather than I_Kr_ playing the major role in repolarizing currents.^1^, ^130^, ^133^ The significant prolongation of repolarization duration, which is affected more by a reduction in I_to_ than a reduction in I_Kr_, underlies the mechanism for heart failure with preserved ejection fraction (HFpEF, typical heart failure symptoms with a normal ejection fraction)‐related ventricular arrhythmias and sudden cardiac death in rodent models.^134^ A rabbit ventricular APD could be shortened, and its plateau could be lost when mouse I_to_ currents were integrated.^1^ Thus, differences in I_to_ densities in different species contribute to variations in the waveforms of action potentials.^1^, ^130^

Kv4.3 is composed of one pore‐forming α subunit and K^+^ channel interacting protein 2 (KChIP2) β subunit^130^, ^133^ and is regulated by several accessory protein interactions (Figure 4B) (Table 8). A reduction in Kv4.3 expression and I_to, fast_ in heart disease, is associated with β‐AR/CaMKII‐mediated activation^133^ and β‐AR/NF‐kB‐mediated activation.^135^ Moreover, an increasing number of studies have speculated that Kv4.3 and Nav1.5 not only regulate each other's functions, but also have the ability to interact with each other.^136^, ^137^ Nav1.5 and Kv4.3 reside is visualized in close proximity (<40 nm) at the membrane.^136^ Overexpression of Kv4.3 protein significantly decreased AP upstroke and Nav1.5 current density without affecting Nav1.5 total protein expression and its kinetic properties.^137^ In addition to auxiliary subunit of KchIP2, Navβ1 subunit also associated with Kv4.3^138^, ^139^ and regulated the I_Na_/I_to_ balance by yielding an increase in I_Na_ and a decrease in I_to_^136^.

**TABLE 8:** Accessory proteins reported to interact with and regulate cardiac Kv

Kv4.3				
Accessory proteins	Types	Binding sites	Biophysical function	Techniques
Dipeptidyl peptidase‐like protein 6 (DPP6)	Additional β‐subunit	Serine proteases but lacks enzymatic activity	Regulates the inactivation and recovery from inactivation properties of Kv4.3 channels. Co‐expression of DPP6 with Kv4.3 and KChIP2 produces a similar current kinetics as in human ventricular myocytes^269^	MPC, WB
SAP97	Anchoring‐adaptor protein	PDZ domain‐binding motif Ser‐Ala‐Leu (SAL) in the CTD	Forms a tripartite complex with CaMKII through SAL motif, increase Ito even if in the absence of KChIP and DDP6^270^	MPC, pull‐down assays
CaMKII	Kinase	SAL motif in the CTD	Forms a tripartite complex with SAP through SAL motif.^270^ A frequency‐dependent reduction in Kv4.3 expression and Ito current is directly linked to increases in CaMKII activation^271^	MPC, Co‐IP

Kv7.1				
Accessory proteins	Types	Binding sites	Biophysical function	Techniques
Lipid phosphatidylinositol 4,5‐bisphosphate (PIP2)	Cofactor	R249 of S4‐S5 linker	In the absence of PIP2, voltage‐sensing domain activation fails to open the pore^272^	MPC, WB
		R190 and R195 of its S2‐S3 loop	Activates Kv7.1 as well as its complexes with different KCNEs^273^	
		Helix B,^274^ helix C^151^ of CTD	LQT mutants in KCNQ1 helix C leads to a decreased current density and a depolarizing shift of channel activation, mainly arising from impaired PIP2 modulation^151^	MPC
A‐kinase anchoring protein 9 (AKAP9)	Anchoring protein	Binds with a leucine zipper (LZ) motif in the CTD	A substrate for PKA phosphorylation by itself, ^275^ and also recruits signaling proteins, such as PKA, PP1 to form macro‐molecular to mediate Kv7.1 phosphorylation or dephosphorylation regulation upon β1‐adrenergic stimulation^146^, ^151^, ^276^, ^277^	MPC, phosphorylation assays, IP
Protein phosphatase 1(PP1)	Protein phosphatase	Recruited via AKAP9	Antagonizes PKA‐mediated S27 phosphorylation^146^	MPC, IP
Calmodulin (CaM)	Regulatory protein	IG in ICL_III‐IV_	Regulates channel gating^144^	MPC
		IQ‐motif in CTD,^152^, ^278^, ^279^, ^280^ a coiled coil formed by the proximal A and B helices, competes with and displaces PIP2 binding at K526 and K527 of helix B in the CTD^274^	Negatively shifts the activation curve^274^	MPC, WB

**TABLE 8:** (Continued)

Kv11.1				
Accessory proteins	Types	Binding sites	Biophysical function	Techniques
14‐3‐3ℇ	Adaptor protein	S283 in NTD and S1137 in CTD	Accelerates channel activation after phosphorylation by β‐AR/PKA, stabilizes and prolongs the phosphorylation state by preventing dephosphorylation^281^	MPC, WB

Kir2.1				
Accessory proteins	Types	Binding sites	Biophysical function	Techniques
AP1‐ γ	Adaptor protein	CTD	Marks Kir2.1 for incorporation into clathrin‐ coated vesicles at the Golgi^174^	Co‐IP, MPC
SAP97	Anchoring‐adaptor protein	NTD, CTD	Regulates surface expression of channels and is assembled a macromolecular signaling complex^282^	Co‐IP, MPC
AKAP79	Anchoring protein	NTD, CTD	Anchors kinase close to channel phosphorylation sites^175^	Co‐IP, MPC
Caveolin‐3	Scaffolding and regulatory protein	NTD	Regulates Kir2.1 trafficking and surface expression^283^	Co‐IP,
PIP2	Cofactor	CTD, NTD	Activates Kir2.1 function^284^	MPC

CTD, carboxyl terminal domain; NTD, N terminal domain; MPC, manual Qpatch clamp; WB: Western blot; co‐IP, coimmunoprecipitation.

Mutations in *KCND3*‐encoded Kv4.3 or *SCN5A*‐encoded Nav1.5 further showed the functional relationship between Kv4.3 and Nav1.5.^136^ GOF and LOF mutations in *KCND3* (Table 9) respectively decreased and increased the Nav1.5 current, respectively.^136^ On the other hand, *SCN5A* LOF mutations increased I_to_ by facilitating Kv4.3 cell surface expression or by slowing its steady‐state inactivation.^136^ Thus, during the early phase of ventricular AP repolarization, a fine balance may exist between I_Na_ and I_to_. GOF mutations in the *KCND3* contributes to increase of peak I_to_ via efficient protein trafficking and gating, resulting in the imbalance of those two currents, the accentuation of the AP notch, and the development of BrS and/or early repolarization syndrome.^136^, ^140^, ^141^, ^142^

**TABLE 9:** Mutations in cardiac voltage‐gated K^+^ channels associated with congenital syndromes

Subtypes	Encoding subunits Gene	Congenital syndrome	Gain or Loss of Function	Mechanisms underlies the phenotype	Examples of variants
Kv4.3	*KCND3*	BrS	GOF	Trafficking efficiency	L450F^136^, ^140^: increases peak I_to_ current density.
				Trafficking efficiency and gating defects	G600R^140^: increases peak Ito current density and slows inactivation.
		ERS	GOF	Gating defects	G306A^141^: significantly increases I_to_ current densities, slows inactivation, and prolongs the recovery from inactivation.
		SUDS predisposing cardiac arrhythmia syndromes	Overlap GOF & LOF, GOF dominance	Gating defects	V392I^142^: increases peak I_to_ current density and total charge, while slows decay time, indicating a BrS‐like I_to_ GOF. While slows the recovery from inactivation. G600R^142^
		Spinocerebellar ataxia (SCA19/22)	LOF	Trafficking defects	∆227F‐Kv4.3^136^
Kv7.1	*KCNQ1*	SQTS2	GOF	Gating defects	F279I^156^: impairs the association with *KCNE1*, produces a negative shift in the activation curve and an acceleration of the activation kinetics leading to increase of I_ks_
					R259H^157^: markedly prolongs the time constant of deactivation leading to a GOF in I_ks_ without affects activation and inactivation kinetics.
		LQTS1	LOF	Trafficking defects	R190Q^149^: leads to a 70% reduction in I_ks._
				Gating defects	D242N^150^: removes the inactivation kinetics, slows the activation kinetics by shifting the voltage dependence of activation to more depolarized potentials thus hindering I_Ks_ current at physiologically relevant membrane potentials.
					A371T and S373P^152^: impairs CaM binding and alters channel assembly, thus stabilizing inactivation, and decreasing current density.
					R555C, R555H, K557E and R562M^151^: markedly reduces the current densities, positively shifts the voltage dependence of activation, slows activation kinetics, increases deactivation rates and reduces interaction with the KCNE1 C‐terminus and PIP2 binding
				Permeation disruption	T322M, T322A, and G325R^153^: cause a complete loss of I_Ks._
	*KCNE1*	LQTS5	LOF	Trafficking defects	L51H^154^
Kv11.1	*KCNH2*	SQTS1	GOF	Gating defects	N588K^265^: increases steady‐state current and abolishes rectification of the current.
		LQTS2	LOF	Synthesis deficiency	Y611H and V822M^266^
				Trafficking defects	80‐90% variants^160^, ^161^, ^162^ cause loss of Kv11.1 expression on plasma surface trafficking to the plasma and exert dominant‐negative effect.
				Gating defects	T421M^267^: positively shifts the voltage dependence of activation. L553N^163^: produces a dramatically faster deactivation time.
				Permeation disruption	G628S^161^, ^266^, ^268^: leads to a reduced selectivity to potassium.
	*KCNE2*	LQTS6	LOF	Gating defects	M54T^164^: increases deactivation rates.
Kir2.1	*KCNJ2*	Borderline for SQTS3	GOF	Gating defects	F58S^187^: produces an increase of the channel conductance and in its open probability
		LQT7 (ATS)	LOF	Trafficking defects	S314/Y315^174^, ^179^: produces reduction of membrane expression reduction and has DN effect on WT.
				Gating defects	V77E/M307V^176^: produces nonconductive Kir2.1 without affecting cell surfaces expression and has DN effect on WT.
				Permeation disruption	V302M^184^, ^185^: disrupts the channel to conduct potassium without altering subunit assembly or suppressing cell surface expression.

LQTS, long QT syndrome; SQTS, short QT syndrome; BrS, Brugada syndrome; ERS, early repolarization syndrome; SUDS, sudden unexplained death syndrome; ATS, Andersen‐Tawil syndrome; GOF, gain‐of‐function; LOF, loss‐of‐function.

#### 4.3.2 Kv7.1

The slow delayed rectifier current, I_Ks_, is mediated by *KCNQ1*‐encoding Kv7.1 and plays an important role in regulating the repolarization phase that terminates cardiac APs and thereby ends contraction (Figure 4C). In the heart, the *KCNE1*, encoding the auxiliary β‐subunit KchIP2, interacts with Kv7.1 α chains and affects both voltage‐sensing S4 movement and the gate,^143^ making the activation of the complex much slower than that of Kv7.1 alone^144^ (Figure 4D). Cryo‐EM analysis revealed a unique feature of Kv7.1: pore opening requires lipid phosphatidylinositol 4,5‐bisphosphate (PIP2) binding during membrane depolarization, thereby increasing current and slowing inactivation.^144^ In addition, Kv7.1 is regulated by accessory protein interactions (Table 8), β‐AR/PKA‐mediated phosphorylation^145^ and PP1‐mediated dephosphorylation^146^ (Figure 4D).

Mutations in *KCNQ1* (Table 9) are the leading cause of several congenital cardiac diseases, including LQTS and SQTS.^147^ LQT1, the most common genotype‐positive LQTS, is associated with LOF mutations in the *KCNQ1*‐encoded Kv7.1 α subunit and is often triggered by β‐AR stimulation.^148^ Trafficking defects,^149^ gating defects,^150^, ^151^, ^152^ or permeation disruption^153^ have been postulated to be the mechanism of decreasing I_Ks_ or hindering I_Ks_ currents at physiologically relevant membrane potentials but limiting the upregulation of I_Ks_ by PKA activation.^145^ Because CaM regulates channel gating by interacting with voltage sensor domains, mutations impair CaM binding (located near the IQ motif of *KCNQ1* C‐terminus) and alter both channel assembly and gating, thus decreasing I_Ks_ current density and contributing to LQT1.^152^ Thus, dysfunction of Kv7.1 caused by *KCNQ* or related accessory protein mutations decreases I_Ks_ or limits the upregulation of I_Ks_ by PKA activation and then contributes to LQT1. I_Ks_ are more sensitive to β‐AR stimulation than I_Kr_.^132^ Enhancement of I_Ks_ by increasing Kv7.1 phosphorylation to shorten the APD during rapid heart rates might represent an effective antiarrhythmic strategy. LOF mutations in *KCNE1* are associated with LQTS5.^154^, ^155^ On the other hand, SQTS2 is associated with GOF mutations in *KCNQ*, which could enhance I_Ks_ via acceleration of the activation kinetics or prolongation of deactivation time constant^156^, ^157^

#### 4.3.3 Kv11.1

I_Kr_ is mediated by Kv11.1, a VGIC encoded by the *KCNH2* gene (also known as the human ether‐a‐go‐go related gene, *hERG*). In cardiac cells, I_Kr_ is rapidly activated during Phase 0 of the AP, followed by rapid inactivation during depolarization in Phase 0‐II. Then, it quickly recovers from inactivation and reopens during the initial Phase III repolarization, followed by slow deactivation that permits sustained Phase III and early Phase IV of the AP (Figure 4E).^158^ Kv11.1 channels exhibit longer‐lasting and higher‐amplitude tail currents than have been found for other outward current channels that contribute to cardiac AP repolarization and duration.^159^

Kv11.1 is composed of one pore‐forming α subunit and two β subunits (MinK and MiRP1 encoded by *KCNE1* and *KCNE2*) (Figure 4F). The structure of the *hERG* channel with depolarized voltage sensors and open pores was revealed using cryo‐EM.^19^ A small central cavity includes extended pockets, which is specific to Kv11.1, explaining the notable susceptibility of this channel to a wide range of drugs.^19^ This high‐resolution structure of the *hERG* channel in the open state also provided the opportunity to investigate the potential mechanisms for the state‐dependent blockade of *hERG* by drugs.^22^

Kv11.1 is regulated by accessory protein interactions and signaling pathways (Figure 4F) (Table 8). Phosphorylation of Kv11.1 could be induced by the stimulation of β‐AR/cAMP/PKA or G protein‐coupled receptors (such as angiotensin II receptor AT1 and the α‐adrenoceptors)/PKC signaling pathway, resulting in a decrease in I_Kr._^158^

LOF mutations in Kv11.1 (Table 9) are characterized by reduced I_Kr_ and are associated with LQTS2, perhaps due to the disruption of the *α* subunits responsible for channel synthesis/translation, a reduction in intracellular transport or the accessory protein interactions required for channel trafficking on the membrane, or the impairment of channel gating structure as well as permeation.^160^, ^161^, ^162^ Among those mechanisms, trafficking defects is the dominant one, responsible for approximately 80‐90% of LQT2 by decreasing the folding efficiency of Kv11.1 proteins and increasing their retention in the endoplasmic reticulum (ER).^160^, ^161^, ^162^ Comprehensive analysis of hundreds LQT2‐linked mutations in four Kv11.1 structural domains and found that deficient protein trafficking is the dominant mechanism for all domains except for the distal C‐terminus. Comprehensive and accurate analysis of mutations between normal and abnormal trafficking across multiple structural domains would aid in understanding the deleterious nature of these mutations.^162^, ^163^ Increasing high‐throughput assays are developing and as alternative to traditional western blot assay to collect functional data.^163^ In addition, LOF mutation in *KCNE2* is associated with LQTS6, a rare type of LQTS.^155^, ^164^ The allosteric modulation (Table 10) of Kv11.1 was investigated to explore methods of alleviating channel dysfunction and increasing I_kKr_ current and may represent a useful new approach for treating inherited and drug‐induced LQTS2.^165^

**TABLE 10:** Kv11.1 modulator with the effects on I_Kr_ current

Modulators	Binding site	Major effect	Side effect	Model	Techniques
VU0405601	Extracellular domain of the Kv11.1 channel rather than to its central cavity	Reduces the APD‐prolonging effect of dofetilide	NR	Primary rabbit hearts	MPC^285^
				HEK293 Kv11.1 cell lines	[3H] dofetilide‐binding assays; radioligand‐binding assays^229^
AZSMO‐23 (types 2 of Kv11.1 activators)	Removal of inactivation	1) Shifts the inactivation curve positively; 2) Increases prepulse and tail current^286^	Blocks hKv4.3‐hKChIP2.2, hCav3.2 and hKv1.5 and activates hCav1.2/β2/α2δ channels	WT/mutation Kv11.1, and other cardiac ion channels expressed cell lines	APC^286^
LUF7244	Strong affinity, allosteric site topologically distinct from where classic Kv11.1 blockers bind	1) Slows rate of deactivation; 2) Shifts the inactivation curve positively; 3) Exerts a significant negative allosteric effect on the binding of typical Kv11.1 blockers; 4) A suppressive effect on proarrhythmia in neonatal rat ventricular myocyte monolayers^229^, ^287^	NR	HEK293 Kv11.1 cell lines	MPC, [3H] dofetilide‐binding assays; radioligand‐binding assays^229^, ^287^
LUF7346	Allosteric site topologically distinct from where classic Kv11.1 blockers bind	1) Slows the rate of deactivation, and 2) shifts activation curve negatively in iPSC‐CMs with the N996I mutation, related to trafficking defect in LQT2^225^	NR	HEK293 Kv11.1 cell lines, iPSC‐CMs from LQT2 patient	MPC, MEAs^225^
Lumacaftor (LUM, clinical drug)	Allosteric site topologically distinct from where classic Kv11.1 blockers bind	Increases channel trafficking on the cell membrane and to reverse field potential duration prolongation in hiPSC‐CMs derived from LQTS2 patients^165^	NR	iPSC‐CMs from LQT2 patient	MEAs, calcium imaging^165^
SB‐335573 (types 4 of Kv11.1 activators, a structural analog of the agonist NS3623	Binding in either open or closed states of channel	Increases the open channel probability	No blocker effect on Cav1.2 and Nav1.5	CHO‐Kv11.1 stable cell line	APC^288^
SKF‐32802 (types 3 of Kv11.1 activators)	Binding in either open or closed states of channel	1) Shifts the inactivation curve positively, and 2) Increases the open channel probability^288^	No blocker effect on Cav1.2 and Nav1.5	CHO‐Kv11.1 stable cell line	APC^288^

APC, automated patch clamp; MEAs, microelectrode arrays; HEK293, human embryonic kidney 293 cells; CHO, Chinese hamster ovary cells.

SQTS1 is caused by GOF mutations (Table 9) in the Kv11.1 channel and is the most prevalent SQTS subtype. Mutations that impair the inactivation of Kv11.1^166^ might explain the lack of efficacy of many class III antiarrhythmic drugs (such as sotalol and ibutilide^167^) in some patients. Interestingly, hydroquinidine, aclass I antiarrhythmic drug inhibiting the Nav1.5 channel, could also block Kv11.1, significantly intervene with ventricular tachyarrhythmia induction^167^ and prolong the QT interval in SQTS patients with Kv11.1 mutations.^168^ In addition, ivabradine, as a class 0 antiarrhythmic drug inhibiting hyperpolarization‐activated cyclic nucleotide‐gated (HCN) channels, could also block I_Kr_ currents by binding in the vicinity of the lipid‐facing surface M651 residue, which is directly coupled to the conformational dynamics of residues in the pore helices,^22^, ^169^ and exert antiarrhythmic effects in SQTS1 hiPSC‐CMs with the N588K mutation.^170^ This represents one important method by which the efficacy of drugs used for SQTS treatment can be evaluated in hiPSC‐CMs with mutant Kv11.1 or multiple ion channels to predict effects in SQTS patients.^171^ It would be worthwhile to further examine the effects of traditional inhibitors and to develop novel specific inhibitors to expand the clinical options available for these patients.

#### 4.3.4 Kir2.1

The strong inward rectifier potassium current I_K1_, primarily mediated by isoforms of the Kir2.x family (*KCNJ2‐*encoding Kir2.1/*KCNJ12‐*encoding Kir2.2), plays a critical role in stabilizing the resting MP and maintaining the duration of the terminal Phase III repolarization in human ventricle myocytes.^38^ Kir2.1 is more dominant than Kir2.2 in human ventricle myocytes^38^ under resting conditions, and Kir2.1 is in an open state and is permeable to K^+^. Kir2.x is abundantly expressed in ventricle and atrial myocytes, but is absent in SAN cells, allowing a relatively depolarized MP and maintaining pacemaker activity in SAN cells.^37^, ^172^ In contrast to adult ventricular CMs, a substantial lack of I_K1_ in hiPSC ventricular CM is regarded as one mechanistic contributor to the immature electrophysiological properties of spontaneous AP. Artificial expression of Kir2.1 might overcome this limitation, render the electrophysiological phenotype to be mature, and ablate proarrhythmic AP traits.^173^

The structure of K_ir_ channels is relatively simple in comparison with Nav, Cav and the members of the voltage‐gated Kv channels. Each subunit of the Kir2.1 tetramer has only two membrane‐spanning helices (S1‐S2) but without the four membrane helices that form the voltage sensor in Kv channels.^174^ Kir2.1 is regulated by accessory protein interactions and signaling pathways (Figure 4F) (Table 8). Newly synthesized Kir2.1 could be sent to specific membrane subdomains for functional expression by Golgi according to a recognition site formed by the residues in the CTD and amino‐terminal domain (NTD) and interaction with adaptor protein complex 1 (AP1).^174^ AKAP79 directly interacts with Kir2.1 through the intracellular N and C domains to promote anchoring other kinases (PKA, PKC) and close to Kir2.1 phosphorylation sites.^175^ PIP_2_ is an essential cofactor for activating Kir2.1 channel function.^176^, ^177^ In addition, Kir2.1 closely interacts with Nav1.5 (Figure 1)^178^, ^179^ and shares a coupled forward trafficking process with Nav1.5.^179^ Normal trafficking of Kir2.1 could enhance the functional expression of Nav1.5 compared to Nav1.5 alone, while trafficking‐deficient variants disrupt Kir2.1 functional expression at the membrane and also exert a DN effect on Nav1.5 functional membrane expression. Thus, in addition to controlling resting MP, I_K1_ could also modify Nav1.5 function and cell excitability. In turn, suppression of Nav1.5 by the CaMKII inhibitor KN93^178^ or by trafficking‐defective Nav1.5 variants could trap Kir2.1 channels,^180^ thus decreasing I_K1_ in addition to I_Na_.

Most *KCNJ2* LOF mutations (Table 9) are associated with type 1 Andersen‐Tawil syndrome (ATS), in which LQTS7 is the primary cardiac manifestation.^181^, ^182^ I_K1_ reduction could prolong the terminal phase of the cardiac AP and contribute to the development of DAD and ventricular arrhythmias in ATS.^183^ LOF mutations could suppress I_K1_ via impairment of PIP2 gating,^176^, ^182^ membrane trafficking,^174^, ^179^ or potassium conduction.^184^, ^185^ On the other hand, GOF mutations in *KCNJ2* (Table 9) cause SQT3.^186^, ^187^

## 5 CARDIOVASCULAR SAFETY EVALUATION

### 5.1 Drug‐induced cardiovascular arrhythmias

In addition to gene mutation‐induced congenital arrhythmias, drug therapy could exert side effects on cardiac VGIC and increase the risk of life‐threatening arrhythmias, such as drug‐induced LQTS (diLQTS) and torsades de pointes (TdPs) that is morphologically distinctive polymorphic ventricular tachycardias with short‐long‐short cycles patten.^3^, ^4^, ^188^ LQT on the surface electrocardiogram correlates with ventricular AP repolarization prolongation at the cellular level.^189^ Drugs can induce AP repolarization prolongation by inhibiting I_Ks_, or, more frequently, I_Kr_. Due to the robustness of I_Kr_, defective I_Ks_ by blockade of Kv7.1 might produce little AP prolongation in humans and other large mammals^190^ but might further prolong AP and induce LQT1 when challenged with β‐AR stimulation^190^ or reduce repolarizing currents by drugs, especially I_Kr_.^191,^ ^192^

Kv11.1 is recognized as a predominant target for diLQTS and TdPs due to its intrinsic arrhythmogenic activity, although it is one of the interests of the development of antitachyarrhythmia drugs. The list of drugs that inhibit Kv11.1 includes not only includes antiarrhythmics (such as dofetilide) but also antipsychotics (such as Pimozide), gastroprokinetic agents (such as cisapride), antihistamines (such as astemizole), and other drug classes.^19^ Among all potassium channels, Kv11.1 is unique in having a small central cavity with extended pockets so that it is susceptible to direct blockade by a wide range of drugs.^19^ In addition, some drugs could exert inhibitory effects on Kv11.1 trafficking^193^ or coexisting effects of channel blocking and trafficking defects, thus causing diLQTS and TdPs.^194^

Due to the increasing attention that diLQTS has attracted from clinics, drug developers, and pharmaceutical regulators,^3^ cardiovascular safety concerns are the most common reasons for the withdrawal of approved drugs from the market or the termination of potential drugs during preclinical or clinical trials.^3^ For example, the noncardiovascular drug cisapride has been withdrawn from the US market because it produces a modest increase in the QT interval in children, causing TdP;^195^ the drug exerts this effect by inhibiting Kv11.1.^196^ Since the outbreak of the coronavirus disease 2019 (COVID‐19), many repurposed drugs are proposed as potential therapies for this disease; their risks, causing LQTS or TdPs is being evaluated.^197^

### 5.2 Development of drug safety evaluation guidelines

Since the guidelines, including the International Council for Harmonisation (ICH) S7B (nonclinical) and E14 (clinical),^198^ were announced in 2005, Kv11.1 channel safety screening data of new drug candidates before beginning clinical trials has become a great need in the pharmaceutical industry.^199^ However, promising drug candidates might be eliminated by the guidelines because variations in the potency of Kv11.1 blocking could result from varying patch clamp protocols and a poor ability to statistically quantify experimental variability.^200^ Moreover, promising drugs might be Kv11.1 blockers but exceptions in terms of causing TdPs or arrhythmia. Some also block other cardiac currents^201^ (Table 11) necessary for TdPs development but do not obviously prolong AP repolarization.^202^, ^203^, ^204^ Thus, in early multichannel studies, a model named multiple ion channel effects (MICE), based on the concentration‐dependent responses of Kv11.1, Nav1.5, and Cav1.2 currents to torsadogenic and nontorsadogenic drugs, was proposed to be more effective than Kv11.1 assays in predicting TdPs.^205^, ^206^ Although the current paradigm has largely kept potential torsadogenic drugs off the markets, but a new cardiac safety paradigm with comprehensive model‐informed approach rather than exclusively by potency of Kv11.1 block and by QT prolongation is urgent to adopted to improve the deficiencies of current paradigm, more specifically discern a real proarrhythmic risk of promising drugs, and enhance the development of effective and safe products or therapeutics.^6^

**TABLE 11:** Agents with multiple channel actions

Drug	Class	Major effect	Effect on other channel	Effect on APD	Techniques	Clinical effect	Current clinical trial
Quinidine	Ia	Nav1.5 open state inhibitor with intermedium dissociation kinetics	Potent Kv11.1 blocker (in S6 segment‐, IC50 = 2μM)^289^	APD prolongation	MPC, APC^289^	Clinical drug available for patients with SQTS^290^	NCT01873950 Phase I completed: Study of the Electrocardiographic Effects of Ranolazine, Dofetilide, Verapamil, and Quinidine in Healthy Subjects
			Kv7.1 blocker	APD prolongation	MPC^145^		
Ranolazine	New class Id	A potent inhibitor of late I_Na,L_	Potent Kv11.1 blocker^204^, ^291^; No effect on Kv11.1 in SQT1 N588K mutation patients^82^, ^291^	Modest APD prolongation	MPC^82^, ^291^; APC^85^	Treatment of angina pectoris^82^	1) NCT01728025 Phase II completed: Long Term Prophylactic Therapy of Congenital Long QT Syndrome Type III (LQT3) With Ranolazine; 2) NCT02133352 Phase IV completed: Treatment of Pulmonary Hypertension Associated with diastolic left ventricular dysfunction; 3) NCT01721967 Phase IV completed: Treatment of Chest Pain in HCM Patients, Hypertrophic Cardiomyopathy; 4) NCT02360397 Phase2 completed: Ventricular Premature Complexes, Myocardial Ischemia; NCT01349491 Phase III terminated: Prevention of Atrial Fibrillation After Electrical Cardioversion
GS‐458967	New class Id	Potent and selective inhibitor of I_Na,L,_ No effect on I_Na,P_ density^292^	Minimal inhibition of I_Kr_, IC50 ratio (I_Kr_ / I_Na,L_ >76 folds)^84^	No prolongation on APD and QRS interval^84^; reduction of APD prolongation in *SCN5A*‐1795insD± hiPSC‐CMs^292^	MPC^84,^ ^292^	NR, potential antiarrhythmic effects^292^	No clinical trial yet
Ivabradine	0	HCN channel blocker	Kv11.1 blocker ^166^, ^169^, ^170^, ^293^ Nav1.5 inhibition^293^	APD prolongation in cardiomyocytes^166^; reverses APD shortening in N588K SQTS1 hiPSC‐CMs^170^; no prolongation of ventricular‐like APs in cardiomyocytes derived from iPSCs^293^	MPC,^293^ single‐cell contraction measurement^170^	Clinical drug available for reduction of heart rate in sinus tachycardia^293^	NCT03866395 Phase IV completed: Ivabradine on Residual Myocardial Ischemia After PCI
Verapamil	IV	L‐type Ca^2+^ channel blocker^20^	Potent Kv11.1 blocker ^294^	No prolongation on APD^294^; decreases the QT interval^203^	MPC and APC^294^	Clinical drug available for heart rate control of atrial fibrillation	NCT01873950 Phase I completed: Study of the Electrocardiographic Effects of Ranolazine, Dofetilide, Verapamil, and Quinidine in Healthy Subjects

HCN, hyperpolarization‐activated cyclic nucleotide.

Clinical trial homepage: https://clinicaltrials.gov.

In 2013, several organizations formed a team to develop the Comprehensive In Vitro Proarrhythmia Assay (CiPA) initiative,^6^ a new paradigm developed with the goal of presenting a deeper understanding of the mechanism of TdPs and improving the assessment of the proarrhythmic effects of potential drugs. It is driven by mechanistically based in vitro assays of drug effects on multiple cardiac channels coupled in silico reconstruction of cardiac AP, and comparison of predicted and observed responses in human‐derived cardiac myocytes. Twenty‐eight drugs with well‐characterized three torsadogenic risk groups (Table S3) have been selected and considered as test cases to build/calibrate model for testing and validation of in silico and stem cell CIPA models.^207^ Several working groups are involved in developing the CiPA:

1. The ion channel group is developing voltage‐clamp protocols by MPC or APC for several key cardiac ion channels. It is believed that at least six ion channels are involved in cardiac APs: Nav1.5, Kv4.3, Cav1.2, Kv11.1, Kv7.1, and Kir2.1.^208^ A study evaluated the predictive ability of these six ion channels using APC and showed that four ion channels provided good predictions, whereas the analysis of three channels wrongfully predicted one high‐risk drug to be safe.^209^ Improved systematic approaches for accurately estimating the potency and safety margins are required.^200^ Increasing APC‐based assays have been explored in Kv11.1,^200^ Nav1.5,^85^ Cav1.2,^210^ Kir2.1,^211^, ^212^ Kv7.1,^212^ and Kv4.3^212^ to improve the evaluation strategies.
2. The in silico group is building computer models to reconstruct electrophysiological activities and drug effects on multiple human cardiac currents by integrating experimental data within a heart cell and subsequently outputting the net impact on the cellular APD and QT interval for predicting drug‐induced proarrhythmic risks.^209^ For example, by using an in silico model, several proposed drugs against COVID‐19 are estimated to have a significant risk for LQTS; thus, mandatory monitoring of the QT interval should be performed among patients in use of drugs.^197^ In silico models are keeping updated to expand the index for discriminating TdPs compounds^213^ and to satisfy a series of general principles for the validation of proarrhythmia risk prediction.^214^ Those principles will help shape the future important directions of more accurate prediction models.^214^ For example, development of better simulating models to capture the drug response not only in normal humans but also in specific patient populations.^214^ With the application of in silico modeling, machine learning could identify cellular electrophysiological phenotypes associated with patients who has certain cardiac diseases and further predict which patients face an elevated risk of ventricular arrhythmias and sudden death.^215^ However, information such as comparisons among drugs with similar chemical or affinity profiles is not yet possible incorporated into in an silico model.^216^ Thus, newer proarrhythmia risk prediction models could be developed to aid in decision making.^216^ For example, a computational pipeline was recently developed to predict Kv11.1 blocker proarrhythmic risk from drug chemistry and distinguish drugs that have similar chemistry and effects on the AP and QT interval but different proarrhythmic risk levels.^216^
3. The myocyte group used iPSC‐CM assays to evaluate the in vitro and in silico assay results.^217^ Native human cardiomyocytes are ideal but with difficulties to obtain, maintain in long‐term culture.^2^ Native cardiomyocytes from different species have variations in the waveforms of APs and drug responses due to differences of potassium currents densities.^1^, ^130^ Rodent is not an appropriate specie for modelling human repolarization due to dominant I_to_; dogs and rabbits are relative closely to human due to the major role of I_Kr_ in repolarization.^218^ Thus, the need for proarrhythmia evaluation in preclinical studies based on human models is emphasized. Currently, hiPSC‐CMs have provided a perfect platform for proarrhythmia evaluation and safety evaluation of human cardiomyocytes in preclinical studies, and various AP parameters could be measured using high‐throughput systems.^15^, ^16^, ^17^, ^30^, ^219^
4. The clinical translation group will use clinical Phase I ECGs to evaluate potential unanticipated effects.

In addition, evaluating the effect of compounds on the overall APs rather than a single ion channel current has been proposed to be a more appropriate approach.^5^, ^6^ However, the more depolarized resting MP of hiPSC‐CMs than that of primary cardiomyocytes is the limitation and challenge of their use in safety evaluation. This is due to the distinct expression level of ion channels expression compared to primary cardiomyocytes, especially the low expression of the I_K1_ channel protein Kir2.1.^173^ Exogenous overexpression of the Kir2.1^173^, ^220^ or electronic injection of an I_K1_‐like current by dynamic clamp into hiPSC‐CMs^221^ to compensate and thus achieve more stable AP facilitates clinical applications, drug discovery, and cardiotoxicity screening. Although it is unclear when the CiPA project will lead to new guidelines (as organizations are generally conservative when considering changes to effective standard protocols), the CiPA initiative and other similar projects worldwide are promoting the development of questions and answers (Q&As) to facilitate the application of the ICH S7B and E14 guidelines.^200^ With the development of these techniques, other cardiac safety liabilities, such as dysfunction of EC coupling and contractile and structural cardiotoxicity, may also be added to electrophysiological tests in the same platform to complement CiPA for regulatory use.^222^, ^223^

In general, with the development of medium‐ or high‐throughput test systems to produce efficient, reliable result output and of basic knowledge of VGICs to update the detection assay designs and analysis methods, drug safety evaluation will receive more attention in preclinical research. Evaluation will be conducted as early as possible to avoid further unnecessary investments in unusable compounds during later stages of drug development.

## 6 CONCLUSION AND PERSPECTIVE

This review provides detailed descriptions of major ion channels in ventricular myocytes, including their expression, structures, regulators, and contributions to normal excitability and congenital pathology. It has been discussed that the application basic and newly discovered knowledge of cardiac ion channels and the continuous development of techniques employed in studies of cardiac ion channels can lead to more attentions to comprehensive proarrhythmic risk assessment in human cardiomyocytes platform in preclinical studies and promote development of cardiovascular safety evaluation guidelines.

Recent research on potential targets of interest in cardiomyocytes, such as TTX sensitive or TTX‐insensitive Nav and Ryr2 regulatory TRICA channels, has opened new avenues for improving our understanding of the molecular mechanisms of Ca^2+^ homoeostasis, EC coupling, and associated cardiac disease pathogenesis. The development of a Nav‐selective inhibitor or a heart‐specific Nav channel‐KO mouse model will be beneficial for further confirming the pathological mechanism of specific Nav channels.^41^, ^43^ The selective inhibition of Nav channels may offer a potential therapeutic target to alleviate arrhythmias during states of Ca^2+^ overload.^41^, ^43^ The development of hIPSC‐CM, high‐throughput techniques for cellular phenotype detection (such as Aps and contraction), computational simulation models facilitate integration of multiple channels, achieving a comprehensive view of channelopathies as a global phenomenon in human myocytes. Modeling of patient‐specific iPSC‐CMs^149^, ^224^ provides great benefit for the precision medicine treatment of congenital cardiac arrhythmia and for the screening of promising or already approved drugs to test for mutation‐specific antiarrhythmic effects.

Over time, technological developments will certainly further promote the study of an increasing number of scientific questions related to cardiac physiology and pathology and reveal additional ion channels with potential involvement. Based on cryo‐EM structures of many VGICs in basic science, a large body of experimental and clinical observations concerning VGICs has been interpreted and summarized by the structural template.^99^, ^100^ The development of clinical and translational medicine could be advanced by the discovery of the potential drug targets within many VGICs, as well as drugs characteristics, targets‐drugs interaction, and computational models for integrating and predicting information. Recently, a novel multiscale approach has been developed to predict drug‐induced arrhythmia directly based on structural models of drug‐channel interactions and kinetics by using integrative experimental and computational modeling and machine‐learning from the atom to the rhythm in the heart.^216^

For potential targets, cryo‐EM structures map and classify hundreds of clinical arrhythmia variants onto all major domains in the structure of many VGICs,^18^, ^21^ reveal the common or distinct clusters of arrythmia mutations among different types of VGICs^99^, ^100^ or different isoforms of the same VGIC,^21^ provide the molecular basis for understanding disease mechanisms, and thus allow the development of structure‐based diagnosis and drug discovery for arrhythmias in the future.^18^ For clinical or potential drugs, the cryo‐EM structure of VGIC‐drug interactions provide structural insights into the binding affinity and mechanism of drugs,^20^, ^22^ which is beneficial for modifying the structure of drugs, screening alternatives or synthesizing new compounds. For example, cryo‐EM structure of Kv11.1 channel in the open state^19^ promotes the investigation of the state‐dependent blockade of Kv11.1 by the heart‐rate‐lowering agent ivabradine,^22^ which could also exert antiarrhythmic effects in SQTS1 hiPSC‐CMs with the N588K mutation.^170^ The development of novel additional pharmacological approaches (eg, activators/allosteric modulators of potassium Kv11.1 and Kv7.1 channels) are needed to counteract both congenital LQTSs, although currently available therapies (implantable cardioverter defibrillators) have yielded good clinical responses.^225^ For example, Lumacaftor, a drug already in clinical use for cystic fibrosis, has been demonstrated to interact with a site distinct from where classic Kv11.1 blockers bind, thereby restoring Kv11.1 trafficking defects and alleviating LQTS2.^165^ Polyunsaturated fatty acids (PUFAs) and their analogs N‐arachidonoyl taurine have been found to speed up Kv7.1 channel opening and restore channel gating of many different mutant channels^226^ PUFAs and their analogs are effective in shortening the cardiac action potential in pharmacologically prolonged ventricular action potential and QT interval in isolated guinea pig hearts^227^ and in hIPSC‐CM.^228^ Therefore, activators of Kv7.1 are also worth developing to treat LQT1 based on structure‐function studies on diverse IKs channel mutations. However, PUFAs analogs vary in selectivity and different effects for Kv7.1, Nav1.5, and Cav1.2 through nonidentical mechanisms. It is necessary to determine the specific binding sites of PUFAs analogs among normal VGICs and to further identify the most therapeutically relevant PUFAs and PUFA analogs in the treatment of different LQTS subtypes. Moreover, if negative allosteric modulators are used in combination with patient‐specific hIPSC‐CM, drugs that have been withdrawn from market or excluded from clinical application due to diLQT effects may be reconsidered or even rescued to clinical use^229^ after safety validation by electrophysiological approaches.

In addition, site‐specific and target‐oriented approaches using nanomaterials (NMs) have been increasingly applied but might exert potential toxicity on ion channels and cardiac electrophysiology.^230^, ^231^ Maybe revealing these NM‐induced structural changes in ion channels could facilitate the modification of bioactive NPs to optimize NM‐based drug delivery and safety.^230^, ^232^

In general, in‐depth studies that combine electrophysiological approaches with other technologies are being used to explore the expression, function, mechanism, and structure, and activity modulation of WT VGICs and a broad variety of mutated VGICs, providing critical contributions to our knowledge of the roles of VGICs in both normal and diseased cardiac functions, thus facilitating to the discovery of potential structurally and functionally guided drug targets for the modification of channel function and for the treatment of inherited or drug induced cardiac diseases, providing a basis for structure‐ and mechanism‐based personalized clinical management, prompting safety control committees to establish more integrated strategies for drug screening, and enabling improved prediction of cardiac risks to provide safer and more effective drugs for clinical use.

## AUTHOR CONTRIBUTIONS

Hua Li, Xiangdong Wang, and Junbo Ge proposed the conception, study design, and had the final approval of the manuscript submitted. Lulan Chen and Yue He participated in the data collections and analysis, the drafting of the manuscript, and the submission.

## COMPETING INTERESTS

There is no conflict of interest involved in this review.

## Supporting information

TABLE S1. Electrophysiological techniques in cardiac VGICs studies
TABLE S2. Joint techniques promoting further researches on characteristics and function of cardiac VGICs
TABLE S3. CiPA compounds
