# Supplementary material for: Ventricular voltage‐gated ion channels: Detection, characteristics, mechanisms, and drug safety evaluation
Source: Clin Transl Med. 2021 Oct 14;11(10):e530. doi: 10.1002/ctm2.530 (PMC8516344; doi:10.1002/ctm2.530)
Supplement: Supplementary file 1 — TABLE S1. Electrophysiological techniques in cardiac VGICs studies TABLE S2. Joint techniques promoting further researches on characteristics and function of cardiac VGICs TABLE S3. CiPA compounds [file CTM2-11-e530-s001.docx]

**Cardiac voltage-gated ion channels: detection, character, mechanism and drug safety evaluation**

# Table S1. Electrophysiological techniques in cardiac VGICs studies

| **Techniques** | **Pros and Cons** |
| --- | --- |
| Manual patch clamp（MPC） technique | During MPC^1^ recording, a glass pipette containing a solution similar to the cytosol is precisely manipulated onto the cell membrane under a microscope, and a giga-ohm (GΩ) seal between the pipette tip and the membrane is then formed by applying slight suction to the pipette. After rupturing the small membrane plug in the pipette tip, electrical signals, such as MP and APs in current-clamp mode or membrane ion channel currents in voltage-clamp mode, can be detected by the whole-cell patch clamp formed by the connection between the pipette and the inside of the cell. By manipulating the MP or together with application of specific channel inhibitors, the individual ion channel currents can be separated from other ion currents. In cardiomyocytes and cell lines, the features of membrane potential (MP), AP amplitudes, AP duration (APD), ion channel current amplitudes, activation/inactivation curves, recovery curves and so on are commonly investigated with MPC.  While, the limitations of MPC are low throughput, laborious, time-consuming, as well as requiring highly trained personnel. |
| Single-channel MPC recording | By using single-channel MPC recording^2^, single-channel properties of cardiac ion channels can also be examined in reconstituted systems by incorporating purified channel proteins into lipid bilayers. This technique is beneficial for control of the membrane and solution compositions on the cis or trans side, and direct evaluation of the ion, peptides on channel function.^3,4^ While, this technique has some disadvantages. First, minor impurities in some mammalian cell preparations might complicate single-channel measurements and the subsequent data.^5^ Moreover, heavy filtration can make distinguishing the characteristics of a specific target channel from other channel subtypes difficult due to impurities in the protein preparation.^6^ Alternatively, purified *E. coli* channel proteins can be used to recapitulate channels with characteristics identical to those produced by native channel preparations from mammalian cells, and therefore, that activities of those channels are unlikely to result from minor impurities in channel protein preparations.^5^ Second, high levels of background noise sometimes occur in ion channel reconstitution experiments with painted lipid bilayers due to their large bilayer capacitance, obscuring the small signals from some channels.^6^ |
| Automated patch clamp (APC) technique | A developed microfabrication technique APC has yielded a revolution in MPC throughput approximately 20 years ago.^7^ In contrast to MPC, in which a pipette is manipulated to seal the cell in top-down mode, APC uses a bottom-up approach and high-throughput (384 or 768) planar recording chip, which is the small aperture placed on the bottom of each well to seal cells.^8^ Cell suspensions are added to the wells of the plate via automated pipettes and cells attached to the aperture by negative pressure to form a GΩ seal for whole-cell recording. The planar recording chips have the ability to record the action potential in the current clamp and ion currents in the voltage clamp mode. The planar recording chips also enable the use of microfluidic channels to maintain the rapid exchange of low-volume solutions around cell recording sites for recording rapidly desensitizing ligand-gated ion channels and testing small quantities of expensive compounds and molecules. The assay for cardiac safety evaluation related Kv11.1 channel was the first one to be established on the newly developed APC instruments and then the increasing need for high-throughput data of this channel in turn to promote advancement of APC system.^9^ Additional features that enable flexibility are also being developed to optimize APC devices, including temperature control, internal/external exchange, average current from multihole recording, optical stimulation, current clamp, and dynamic clamp.^8,10^ Cell lines transfected with ion channels and hiPSC-CMs are suitable for APC recording.^11^ In contrast to preparations of primary cardiomyocytes hiPSC-CMs or commercially available cardiomyocytes are become a viable option for safety screening. Primary cardiomyocytes are usually to be contaminated by fibroblasts which would reduce the successful rate and accuracy of sealing primary cardiomyocytes under APC. In addition, the irregular morphology and strong contractility of primary cardiomyocytes isolated from adult animal models also affect the stable sealing and recording. Primary cardiomyocytes isolated from younger animal models may be easier to seal and record, but the immaturity of channel expression may lead to biased results. The combination of APC and hiPSC-CMs help the pharmacology community acquire more comprehensive validation data.^11^ In addition, many high-quality cells and careful internal/external preparation are required to improve success rate and to ensure accurate results. The generation and development of novel uses for APC have been accelerated by cooperation between APC manufacturers and academic researchers, in the form of experimental protocols and troubleshooting guidance.  While, APC are limited to large number of isolated cells from suspensions and thus not suitable for studying the processes involved in synaptic communication.^12^ Moreover, the cell cost is also a concern for primary, rare and expensive cells, such as hiPSC-CMs. Optimizing protocol to harvest more cells^13^ or minimizing cell usage for APC could improve cost efficiency and raise the limited availability for primary cells. ^14^ |
| Microelectrode arrays (MEAs) | Cardiac tissue from animal ventricles, isolated cardiomyocytes or hiPSC-CMs^15-17^ can be plated in the wells of MEA plates, which contain an array of embedded substrate-integrated extracellular electrodes on the bottom of the well. The cleft is then filled with an ionic solution between cells and substrate electrodes and is regarded as the seal resistance, and voltages can be recorded. Notably, the interval from the initial sharp deflection to the later moderate return-to-baseline deflection of the field potential is regarded as field potential duration (FPD)，which is closely related to QT interval on ECGs.^18,19^  MEAs have limitations such as a relatively low amplitude resolution compared the patch clamp^20^, FP shape variation due to the difference in the protocols of experimenters^18,19^ (including electrode specification, synchronous degree of cells), and electrical artifacts evoked by stimulation. |
| Impedance | Impedance has been recently applied in electrophysiological assays as a noninvasive, easy-to-use, label-free, high-throughput and long-term measurement strategy for assessing cell proliferation, cardiomyocyte mechanical contractility, and drug candidates proarrhythmogenic probability without altering cellular physiology.^21-23^ Several models, including hiPSC-CMs, embryonic stem cells, primary CMs and stable cell lines could be used in impedance. CardioExcyte 96 recordings with one gold recording electrode can provide a combined readout of synchronous beating and contractility of monolayer cardiomyocytes (via impedance) and electrophysiology endpoints (via EFP) at physiological temperature.^22,23^ For example, an hERG blocker was found to decrease the beat rate and prolong the field potential duration, while isoproterenol and nifedipine were found to increase the beat rate in impedance and EFP modes.^22,24^ |

# Table S2. Joint techniques promoting further researches on characteristics and function of cardiac VGICs

| **Techniques** | **Pros and Cons** |
| --- | --- |
| Cryo-Electron Microscopy (Cryo-EM) | Cryo-EM provides high-resolution structural analyses at less than 10 Å level. An accurate and comprehensive structural model of specific ion channel would facilitate the discovery of drugs with promising targets.^25,26^  While, some questions relating to ion channel structure have not yet been determined by cryo-EM. First, Protein isolation and purification are remaining big challenges for some VGICs, thereby they have not been completely defined as full-length Cryo-EM structure^27^, or even have not been revealed yet. Because some components will be lost during protein isolation and purification due to the weak affinities between individual proteins or the dissociation of bridging component.^28^ Or it might be due to aggregation during purification, or the low expression level to obtain sufficient purified cryo-EM samples. Nowadays, some alternative approaches have been employed to try to address this issue. For example, truncated constructs of hERG channel was used to avoid the aggregation.^29^ Additionally, a Nav1.7 mutation (E406K) was found to result in 3-fold higher expression than WT Nav1.7, enabling the collection of sufficient samples.^30^ Third, the fast transitions between the different voltage-dependent states of ion channels can complicate structural analysis, as they are associated with rapid conformational changes, rearrangement of interactions among adjacent/distant structural elements or limitations of the sample preparation conditions. Fourth, the structures of VGICs in multiple functional states must also be identified to precisely elucidate the mode of agonists and antagonists, as structurally similar molecules may still exert opposite effects on channels.^26^ For example, both antagonist nifedipine and agonist Bay K8644 belong to DHP molecules and occupy the same site of Cav1 channel, while nifedipine is more favored in the inactivated state of Cav1 than Bay K8644.^26^ |
| Optical imaging | ArcLight-expressing hiPSC-CMs showed changes in fluorescence levels as a result of the cardiac AP cycle, with a reduction in intensity during membrane depolarization followed by an increase during membrane repolarization and the resting state.^31^ GCaMP5G-expressing hiPSC-CMs exhibited increased fluorescence intensity of intracellular calcium levels during systole and a reduction in fluorescence intensity during relaxation.^32^ Potential side effects of GCaMP, such as interference with CaM/LTCC gating and signaling and potential cardiotoxicity that induces hypertrophy upon long-term usage, have also been reported.^33^ GCaMP-X, an optimized form of GCaMP, was designed to resolve these limitations for future systems.^33^ The similar results could be achieved using ArcLight optical recordings in comparison with patch clamp. Optical APD90 was significantly longer in disease hiPSC-CMs than in control hiPSC-CMs using ArcLight optical recordings, which is consistent with results from the same cells by patch clamp.^16^  While, those indicators are still needed to be optimized to avoid the potential side effects on cardiotoxicity upon long-term usage.^33^ |
| Optogenetics | Light-activated ion channels, such as channelrhodopsin-2 (ChR2) was first expressed in mammalian neurons to modulate neuronal excitability in 2005.^34^ A wavelength of approximately 470 nm opened ChR2, allowing an inward current to depolarize the membrane and elicit an AP by stimulating the opening of Nav channels. Since then, ChR2 and other light-activated proteins have become popular in various experimental capacities. By using light to control the opening of these channels, specific ions can be allowed to move across the membrane in a controlled manner, depolarizing or hyperpolarizing the membrane to cause cellular excitation or inactivation, respectively. Optical perturbation of membrane voltage has enabled electrophysiology studies in systems that previously could not be analyzed using electrodes.^35^ Notably, the pacing of cardiomyocytes expressing ChR2 can be modulated by applying different light frequencies, allowing the precise modulation of cellular activity and enabling the precise stimulation of cardiomyocytes and cardiac tissue by light instead of electrical stimuli, which often induce electrical artifacts and variation due to nonspecific stimulation.^36^  It is now possible to position LED arrays above the cell plate without affecting impedance and field potential, or position LED arrays below a patch clamp chip without affecting pipetting and internal/external solution exchange, further provide possibilities for high-throughput research and drug screening. An LED lid containing 96 high-power 470 nm LEDs to ensure the illumination of each well of the sensor plate was built in CE96.^37^ Because light-activated proteins expression via AAV is stable for a long time, optogenetics can be employed to examine the chronic effects of drugs. Thus, optogenetics provides an invasive and precise stimulus to modulate the cell excitability, and are beneficial for patch clamp to examine the functional change of ion channel during light stimulus. |

# Table S3. CiPA compounds

| **TdP Risk Level** | **High TdP Risk** | **Intermediate TdP Risk** | **No or Very Low TdP Risk** |
| --- | --- | --- | --- |
| Compounds | Azimilide  Bepridil  Dofetilide  Ibutilide  Quinidine  Vandetanib Disopyramide*  Sotalol | Astemizole Chlorpromazine Cisapride Clarithromycin Clozapine Domperidone Droperidol Terfenadine  Pimozide  Risperidone Ondansetron | Diltiazem  Loratadine  Metoprolol  Mexiletine  Nifedipine  Nitrendipine  Ranolazine  Tamoxifen  Verapamil |

CiPA homepage: https://cipaproject.org

*As of May 10, 2016

# References

1. Neher, E. & Sakmann, B. Single-channel currents recorded from membrane of denervated frog muscle fibres. *Nature* **260**, 799-802 (1976).

2. Fozzard, H.A., January, C.T. & Makielski, J.C. New studies of the excitatory sodium currents in heart muscle. *Circ Res* **56**, 475-485 (1985).

3. Wang, X.H.*, et al.* Structural basis for activity of TRIC counter-ion channels in calcium release. *P Natl Acad Sci USA* **116**, 4238-4243 (2019).

4. Zhou, X.*, et al.* TRIC-A Channel Maintains Store Calcium Handling by Interacting With Type 2 Ryanodine Receptor in Cardiac Muscle. *Circ Res* **126**, 417-435 (2020).

5. Weisleder, N., Takeshima, H. & Ma, J. Immuno-proteomic approach to excitation--contraction coupling in skeletal and cardiac muscle: molecular insights revealed by the mitsugumins. *Cell Calcium* **43**, 1-8 (2008).

6. Pitt, S.J.*, et al.* Charade of the SR K+-Channel: Two Ion-Channels, TRIC-A and TRIC-B, Masquerade as a Single K+-Channel. *Biophys J* **99**, 417-426 (2010).

7. Sigworth, F.J. & Klemic, K.G. Patch clamp on a chip. *Biophys J* **82**, 2831-2832 (2002).

8. Milligan, C.J. & Moller, C. Automated planar patch-clamp. *Methods Mol Biol* **998**, 171-187 (2013).

9. Kiss, L.*, et al.* High throughput ion-channel pharmacology: planar-array-based voltage clamp. *Assay Drug Dev Technol* **1**, 127-135 (2003).

10. Obergrussberger, A.*, et al.* An update on the advancing high-throughput screening techniques for patch clamp-based ion channel screens: implications for drug discovery. *Expert Opin Drug Discov* **13**, 269-277 (2018).

11. Moller, C. & Witchel, H. Automated electrophysiology makes the pace for cardiac ion channel safety screening. *Front Pharmacol* **2**, 73 (2011).

12. Py, C.*, et al.* From understanding cellular function to novel drug discovery: the role of planar patch-clamp array chip technology. *Front Pharmacol* **2**, 51 (2011).

13. Li, W.*, et al.* Establishment of an automated patch-clamp platform for electrophysiological and pharmacological evaluation of hiPSC-CMs. *Stem Cell Res* **41**, 101662 (2019).

14. Becker, N.*, et al.* Minimized cell usage for stem cell-derived and primary cells on an automated patch clamp system. *J Pharmacol Toxicol Methods* **68**, 82-87 (2013).

15. Shinozawa, T.*, et al.* Recapitulation of Clinical Individual Susceptibility to Drug-Induced QT Prolongation in Healthy Subjects Using iPSC-Derived Cardiomyocytes. *Stem Cell Reports* **8**, 226-234 (2017).

16. Itzhaki, I.*, et al.* Modelling the long QT syndrome with induced pluripotent stem cells. *Nature* **471**, 225-U113 (2011).

17. Navarrete, E.G.*, et al.* Screening Drug-Induced Arrhythmia Events Using Human Induced Pluripotent Stem Cell-Derived Cardiomyocytes and Low-Impedance Microelectrode Arrays. *Circulation* **128**, S3-U51 (2013).

18. Meyer, T., Leisgen, C., Gonser, B. & Gunther, E. QT-screen: high-throughput cardiac safety pharmacology by extracellular electrophysiology on primary cardiac myocytes. *Assay Drug Dev Technol* **2**, 507-514 (2004).

19. Meyer, T., Boven, K.H., Gunther, E. & Fejtl, M. Micro-electrode arrays in cardiac safety pharmacology - A novel tool to study QT interval prolongation. *Drug Safety* **27**, 763-772 (2004).

20. Spira, M.E. & Hai, A. Multi-electrode array technologies for neuroscience and cardiology. *Nat Nanotechnol* **8**, 83-94 (2013).

21. Peters, M.F., Lamore, S.D., Guo, L., Scott, C.W. & Kolaja, K.L. Human stem cell-derived cardiomyocytes in cellular impedance assays: bringing cardiotoxicity screening to the front line. *Cardiovasc Toxicol* **15**, 127-139 (2015).

22. Obergrussberger, A.*, et al.* Safety pharmacology studies using EFP and impedance. *J Pharmacol Tox Met* **81**, 223-232 (2016).

23. Doerr, L.*, et al.* New easy-to-use hybrid system for extracellular potential and impedance recordings. *J Lab Autom* **20**, 175-188 (2015).

24. Bot, C.T.*, et al.* Cross - site comparison of excitation-contraction coupling using impedance and field potential recordings in hiPSC cardiomyocytes. *J Pharmacol Tox Met* **93**, 46-58 (2018).

25. Pan, X.*, et al.* Structure of the human voltage-gated sodium channel Nav1.4 in complex with beta1. *Science* **362**(2018).

26. Zhao, Y.*, et al.* Molecular Basis for Ligand Modulation of a Mammalian Voltage-Gated Ca(2+) Channel. *Cell* **177**, 1495-1506 e1412 (2019).

27. Jiang, D.*, et al.* Structure of the Cardiac Sodium Channel. *Cell* (2019).

28. Wu, J.*, et al.* Structure of the voltage-gated calcium channel Ca(v)1.1 at 3.6 A resolution. *Nature* **537**, 191-196 (2016).

29. Wang, W.W. & MacKinnon, R. Cryo-EM Structure of the Open Human Ether-a-go-go-Related K+ Channel hERG. *Cell* **169**, 422-+ (2017).

30. Shen, H., Liu, D., Wu, K., Lei, J. & Yan, N. Structures of human Nav1.7 channel in complex with auxiliary subunits and animal toxins. *Science* **363**, 1303-1308 (2019).

31. Leyton-Mange, J.S.*, et al.* Rapid Cellular Phenotyping of Human Pluripotent Stem Cell-Derived Cardiomyocytes using a Genetically Encoded Fluorescent Voltage Sensor. *Stem Cell Reports* **2**, 163-170 (2014).

32. Shinnawi, R.*, et al.* Monitoring Human-Induced Pluripotent Stem Cell-Derived Cardiomyocytes with Genetically Encoded Calcium and Voltage Fluorescent Reporters. *Stem Cell Reports* **5**, 582-596 (2015).

33. Yang, Y.X.*, et al.* Improved calcium sensor GCaMP-X overcomes the calcium channel perturbations induced by the calmodulin in GCaMP. *Nat Commun* **9**(2018).

34. Boyden, E.S., Zhang, F., Bamberg, E., Nagel, G. & Deisseroth, K. Millisecond-timescale, genetically targeted optical control of neural activity. *Nature Neuroscience* **8**, 1263-1268 (2005).

35. Cohen, A.E. & Venkatachalam, V. Bringing bioelectricity to light. *Annu Rev Biophys* **43**, 211-232 (2014).

36. Bruegmann, T.*, et al.* Optogenetic control of heart muscle in vitro and in vivo. *Nat Methods* **7**, 897-900 (2010).

37. Rehnelt, S.*, et al.* Frequency-Dependent Multi-Well Cardiotoxicity Screening Enabled by Optogenetic Stimulation. *Int J Mol Sci* **18**(2017).
